# Economic implications of autonomous adaptation of firms and households in a resource-rich coastal city

**DOI:** 10.1038/s41598-023-46318-2

**Published:** 2023-11-21

**Authors:** Alessandro Taberna, Tatiana Filatova, Stefan Hochrainer-Stigler, Igor Nikolic, Brayton Noll

**Affiliations:** 1 Department of Multi Actor Systems, Delft University of Technology; Faculty of Technology, Policy and Management, Jaffalaan 5, 2628BX Delft, The Netherlands; 2 International Institute for Applied Systems Analysis, Schlossplatz 1, 2361 Laxenburg, Austria

**Keywords:** Climate-change adaptation, Climate-change impacts, Environmental economics

## Abstract

Climate change intensifies the likelihood of extreme flood events worldwide, amplifying the potential for compound flooding. This evolving scenario represents an escalating risk, emphasizing the urgent need for comprehensive climate change adaptation strategies across society. Vital to effective response are models that evaluate damages, costs, and benefits of adaptation strategies, encompassing non-linearities and feedback between anthropogenic and natural systems. While flood risk modeling has progressed, limitations endure, including inadequate stakeholder representation and indirect risks such as business interruption and diminished tax revenues. To address these gaps, we propose an innovative version of the *Climate-economy Regional Agent-Based* model that integrates a dynamic, rapidly expanding agglomeration economy populated by interacting households and firms with extreme flood events. Through this approach, feedback loops and cascading effects generated by flood shocks are delineated within a socio-economic system of boundedly-rational agents. By leveraging extensive behavioral data, our model incorporates a risk layering strategy encompassing bottom-up and top-down adaptation, spanning individual risk reduction to insurance. Calibrated to resemble a research-rich coastal megacity in China, our model demonstrates how synergistic adaptation actions at all levels effectively combat the mounting climate threat. Crucially, the integration of localized risk management with top-down approaches offers explicit avenues to address both direct and indirect risks, providing significant insights for constructing climate-resilient societies.

## Introduction

The effects of climate change are increasing the frequency and severity of floods worldwide, particularly in coastal areas^1^. As a result, the likelihood of experiencing consecutive severe flood events increases. This necessitates the exploration of cascades and non-linearities in complex economic systems, which, when interacting with socio-economic dynamics, can amplify such risks. Scholars and policymakers recognize that taking anticipatory action involving actors from all levels of society offers the best chance to address this growing threat^2^.

Traditionally, climate change adaptation (CCA) studies have primarily focused on government-led actions, such as the construction of dykes or levees^3^. More recently, there has been an increased emphasis on individual actions, though this has been mainly restricted to households^4^. Unfortunately, the predominant focus on single protective measures, whether initiated by the government or households, overlooks how various measures deployed by different actors might align or conflict^5^. Empirical evidence demonstrates that inadequately coordinated actions can lead to unforeseen consequences triggering feedback that exacerbates existing flood risks^6,7^. Nonetheless, the direct and indirect influence of individual adaptation decisions on the overall system resilience remains largely uncharted territory. This knowledge gap complicates the design of adaptation strategies that are both efficient in managing flood risks and effective in mitigating the associated socio-economic fallout.

Present macro models for quantifying flood risks rely on a static approach, failing to incorporate the complexity of path dependency and non-linear interactions between environmental and anthropogenic systems^8^. The latter although often adept at managing a singular hazard, might struggle to maintain resilience under the accumulating stress of repetitive or consecutive shocks. Thus, acknowledging and incorporating these intricate dynamics into policy models is a pivotal step toward designing more robust strategies despite escalating climatic risks and for constructing an accurate appraisal of damage and adaptation behavior across varied socio-economic strata^9,10^.

Agent-Based Models (ABMs) have emerged as a useful method for modeling heterogeneity, learning, interactions, and out-of-equilibrium dynamics^11–13^. ABMs are versatile in simulating climate change^14–17^ and disaster scenarios^18,19^, flooding in particular^20,21^. Hence, ABMs are particularly useful for capturing evolutionary non-linear and path-dependency phenomena, such as feedback and ripple effects stemming from the interplay of agglomeration economies and a worsening climate^22^. However, flood-ABMs still have limitations, such as a focus on households and neglect of the role of firms and indirect losses resulting from business interruption, loss of employment opportunities, and tax revenues. To address these limitations, we developed a novel version of the *Climate-economy Regional Agent-Based* (CRAB) model^22,23^ that includes bottom-up and top-down adaptation strategies.

CRAB models the socio-economic dynamics of regional agglomeration economies, with a growing concentration of people and assets confronting climate-driven risks. The model features a flood-prone regional economy, consisting of varied households and firms that interact, acquire knowledge, and autonomously decide on actions, such as deciding production quantities, adaptation measures, or possible relocation. This novel version features a layered risk framework that includes individual disaster risk reduction measures, insurance, and government subsidies^24^.

The endogenous agglomeration process stemming from the CRAB model facilitates the exploration of the reciprocal relationship between anthropogenic activities, such as the rising population in flood-prone regions, and climate-induced risks. Introducing severe flood events within a brief period to the regional economy makes it highly suitable to study how localized compounding climate shocks create a feedback loop with socio-economic dynamics influenced by human actions. Furthermore, by integrating diverse CCA actions available to agents, the model provides a powerful lens through which we can observe how individual responses can bolster the resilience of socio-economic systems in the face of escalating risks.

The options for risk reduction and management vary based on the severity and frequency of a disaster. Catastrophe modeling methodologies often utilize a loss distribution, associating losses with their respective probabilities, which enables a comprehensive assessment of these options, including the incorporation of possible changes in future risks due to climate and global change. These loss distributions can be divided into distinct risk-layers^25^. Typically, the low-risk layer encompasses frequent events that can be managed through  risk reduction strategies. In the context of flooding, these strategies often involve individual measures aimed at damage reduction, such as home elevation or flood barriers. For the medium-risk layer, when risk reduction becomes cost-prohibitive, consideration shifts towards risk-financing options like insurance. Flood insurance typically includes entry and cut-off points determining the deductible and unreimbursed damages beyond a certain limit. The high-risk layer, which includes events resulting in extreme losses, is either treated as a residual risk or covered by external assistance or global funding schemes^25^. Since losses must be financed, considerable opportunity costs may be borne by those directly affected by a disaster, such as households, businesses, and the government, as well as by indirectly affected entities (e.g., business interruption)^26^. The risk-layer approach usually omits these indirect damages. Yet, they can be substantial, occasionally exceeding the direct losses^24^. Thus, for indirect risk management, a variety of strategies may need to be considered. This is especially pertinent for CCA where individual risk reduction measures might efficiently mitigate damages from a single hazard but could falter under repetitive shocks, resulting in damage accumulation across various actors and scales. The exploration of multi-scale adaptation options for reducing risks shaped by both direct and indirect damages is rarely done.

The aim of this study is to explore the compound risks stemming from the complex interplay between endogenous economic growth,  repetitive climate-induced shocks causing direct and indirect damages, and multi-scale adaptation. Here we focus on floods as an emblematic climate-induced hazard, which accelerating severity and probability threatens the development of coastal urbanized regions. In order to analyze the interactions across different measures undertaken by various stakeholders, we incorporate multiple protective CCA strategies by households, as well as insurance options available to both households and firms. These are complemented by government-led subsidies aimed at supporting individual CCA measures. The emphasis of this study is not merely on the isolated effectiveness of each individual action but rather on the cumulative protection that these actions can provide. We are particularly interested in examining how different individual actions may interact with one another and with top-down government financial support, to create a more comprehensive and nuanced understanding of adaptation strategies. Hence, we did not include top-down public CCA measures such as dikes and levees, whose effect has been already extensively studied in the existing socio-hydrology literature^1,27^. In particular, we address the following research questions: 1) How do different private CCA strategies impact regional economic growth and fiscal stability in the face of extreme flooding events? 2) To what extent does a government subsidy affect the distribution of bottom-up CCA uptake? How does it impact households with varying adaptive capacities, and what are the resulting direct and indirect damages? 3) What is the regional consequence of inaction for an agglomeration economy hit by extreme flooding? How do these damages  change when top-down subsidies are complemented with bottom-up CCA measures?

To shed light on these questions, we calibrate households’ behavior and socio-economy characteristics using rich survey data from the greater Shanghai area^28^. While our model is inspired by the characteristics of a Chinese coastal urban setting, it primarily represents an archetype of a resource-rich city, as defined by the IPCC^2^. Our aim is not to emulate a specific urban landscape, but rather to extract overarching insights about the interplay and efficacy of various CCA strategies and their distributional impacts. These insights are targeted at coastal urban areas confronting both population and asset growth, while simultaneously facing the growing threats of sea-level rise and flooding. Specifically, we aspire to contribute to the area of economic modeling of adaptation that lacks the exploration of market-based public adaptation policies (e.g. subsidies). Moreover, while empirical research on adaptation (e.g. surveys, interviews, participatory engagement and ethnographic work) provides important data, which increasingly enters physical damage assessments^29^, understanding private adaptation and its dynamics remains one of the key areas of future research^4^ and modeling wider economic consequences of private adaptation is nearly non-existent^30^.

The results emerging from state-of-the-art simulation modeling and survey data on household CCA, show how single adaptation strategy in isolation is ineffective and fails to curb compounding risks. Instead, the combination of CCA actions at all levels — top-down government subsidies, bottom-up protective measures from households, and insurance from households and firms — can produce climate-resilient long-term economic growth and development. When accounting for non-linear effects in the economic response to hazards, government subsidies for individual CCA actions appear cost-effective as they not only reduce direct damages but also create indirect co-benefits like decreasing public spending in unemployment subsidies and sustained regional tax revenues from firms which did not get bankrupt in the aftermath of a disaster and could continue contributing to the economic development of the region. In particular, top-down subsidies show particular effectiveness in the case of consecutive hazard events and in providing financial resources to the most vulnerable individuals. However, due to a lack of resources and opportunities, the latter remains the most exposed to direct and indirect risk, highlighting the possible presence of poverty traps.

## Coastal regional economy with consecutive floods and adaptation

The CRAB is an evolutionary economic ABM designed to delve into the intricate interplay between agglomeration forces — those clustering people and assets along coastlines and delta rivers — and climate-induced shocks^22,23^. It features a regional economy exposed to flooding that encompasses four classes of a variable number of agents: households, capital-good firms, consumption-good firms, and consumption-service firms. Each of these agents is underpinned by unique behaviours and decision-making processes. The capital-good firms, for instance, are the innovation vanguards, channelling investments into R &D with the aim of discovering new and more productive technologies, subsequently driving the “Schumpeterian” innovative destruction process and fueling endogenous economic growth. These technological advancements are then sold as machineries to other firms in a decentralized capital market (for detailed information on agents’ characteristics and interactions see SI; “Model Complements”, “Methods”).

**Figure 1:**
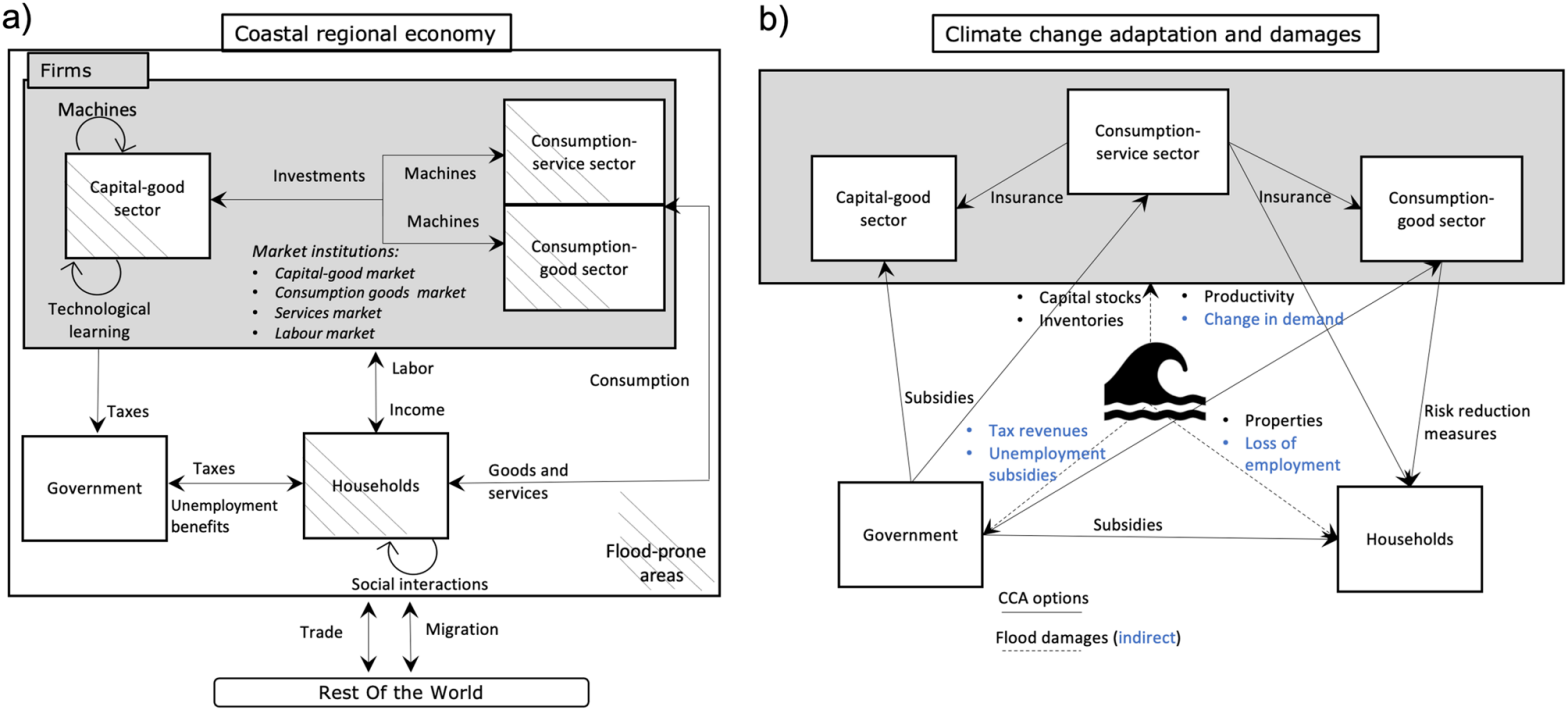
A conceptual representation of the CRAB model regional economy (Panel** a**), and a schematic representation of climate change adaptation actions and damages among agents (Panel **b**).

The modelled regional economy resembles a coastal archetype of a resource-rich megacity. As a representative example of such archetype, we employ hazard, economic and behavioural data from Shanghai, China. The presented results do not aim to predict the adaptation pathways for this city specifically, and hence could omit case-specific details. Instead, we aim to identify generic dynamics in an economic system driven by agglomeration forces, increasing damages and adaptation needs and capacities of different actors. Specifically, our intent is to simulate a regional economy characterized by an influx of population stemming from job opportunities, investments, and economic growth. Economically attractive till recently, the growth in coastal regions is now threatened by accelerating floods and sea-level rise. Notably, in our archetype, the majority of agents are both endowed with the necessary resources and have the intrinsic motivation to employ adaptation strategies that mitigate the adverse effects of floods. The utilization of an archetype-driven approach allows us to identify general patterns in climate change adaptation and a comprehensive understanding of their interaction with complex systems. Thus, by focusing on these general patterns, we strive to derive insights applicable to similar regions, enabling an exploration of both public and private adaptation strategies in resource-rich coastal cities.

To achieve our goal, we use survey data from the greater Shanghai area to calibrate household socioeconomic and behavioural characteristics^28^. We included 10000 properties created from the survey data, which is about 0.2% of the total owner-occupied units in the region. Additionally, we calibrate the initial number of firms according to the current business-to-population ratio. We employ Shanghai flood maps to calibrate the flood depth for each agent for a 10, 100, and 1000 return period^31^. The maps include low- and high-end climate scenarios (RCPs 2.6 and 8.5), which affect flood depth, whose level changes in the years 2030, 2050, and 2100. The amount of damage for each agent is calculated by overlaying flood depth with building class-specific depth-damage curves of Shanghai^32^. Furthermore, we employ national statistics to divide household expenditure between goods and services. The capital intensity of our macro sectors is calibrated by applying constant capital-output ratios from China^33^. Regarding the remaining parameters, we utilize an indirect calibration approach, adjusting them to match specific stylized facts in line with our intent^34 ^(for more information about model calibration see, SI; “Regional economy”). Importantly, following this calibration approach, we acknowledge the possibility of equifinality, recognizing that multiple pathways might lead to similar outcomes in a complex model.

Each step of the CRAB model corresponds to one quarter and we run the model for 300 steps, which is 75 years (2005-2080, where the first 15 years are used as a warm-up period). Floods of 10 years return happen randomly in the model. However, given the importance of studying catastrophic climate change scenarios^35,36^, we consider two fixed floods, namely a 1000-year return at time 200 and a 100-year return at time step 240, that correspond to the year 2055 and 2065 respectively. While a fully stochastic approach was initially considered to capture the entire range of potential flood events, it was rendered unfeasible due to computational limitations. Nevertheless, this design choice allowed us to contextualize regular flood events and their interactions with autonomous adaptation mechanisms against the compound effects of extreme flood events that manifest consecutively. The comparison with a baseline scenario where no floods occur provides a clearer understanding of the differences in both impacts and how people adapt.

Our initial experiment investigates the efficacy of various top-down and bottom-up CCA actions available to our agents. The five scenarios considered are: ‘None’ (indicating no adaptation measures are taken), ‘Insurance’ (only insurance is implemented), ‘DR’ (only protective household measures are implemented), ‘DR & Insurance’ (both protective household measures and insurance are implemented), and ‘Subsidy & DR & Insurance’ (public subsidy is added to finance private measures alongside protective household measures and insurance). We evaluate the economic performance of each scenario in response to floods by analyzing the GDP growth and the fiscal implications, specifically the deficit to GDP ratio. Additionally, we compare the short- and long-term regional dynamics of the CRAB model under mild and worsening climate conditions (RCP 2.6) and under more extreme climate conditions (RCP 8.5) to simulate possible trajectories of global GHG emissions. Despite some differences in magnitude, the qualitative results of the CRAB model simulations under RCP 2.6 and RCP 8.5 are generally similar. However, the impacts of more extreme climate conditions (RCP 8.5) are more evident and marked in the model outputs. As a result, we have chosen to focus our analysis on the RCP 8.5 scenario in the main text. This approach allows us to more clearly highlight the risk of an extreme climate ‘endgame’ scenario^36^.

## Results

### Climate change adaptation uptake, economic growth and fiscal stability

The simulation results indicate that prior to the occurrence of the severe 1000-year flood in 2054, the bottom-up CCA measures available in each scenario are widely adopted even without top-down incentives. Specifically, ‘Wet-proofing’ and ‘Dry-proofing’ are adopted by approximately 50-60% of households at risk, while ‘Elevation’ is adopted by 30-40%, which is expected given the higher cost and implementation difficulties. Additionally, more than 70% of eligible households have insurance coverage. The high adoption rates are attributable to the socio-economic and behavioral characteristics of the population residing in the region. In particular, the majority of households in Shanghai, as in other resource-rich coastal megacities^28^, possess both the willingness and necessary resources to implement protective measures (see Fig. S3 and Table S3 - in SI).

**Figure 2:**
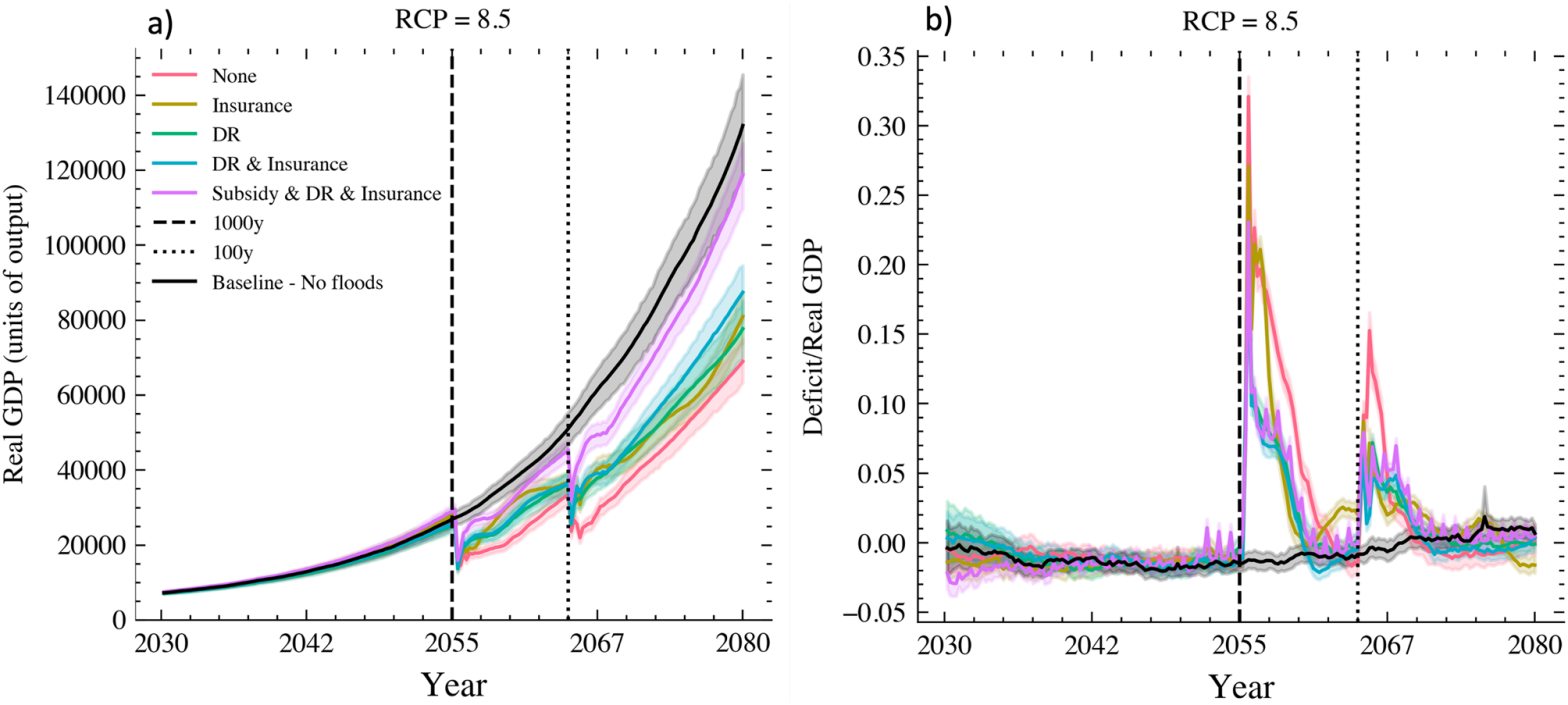
Panel (**a**) shows the average economic growth while panel (**b**) displays the deficit to GDP ratio of the regional economy under each CCA scenario. The reported values are results of a stress-testing under the high-end RCP 8.5 scenario, and averaged across the 100 Monte Carlo runs with the shaded areas denoting the standard deviations. The vertical lines represent the 1:1000 and 1:100 floods. Deficit (*D*) at time *t* is defined as the net of cost and revenues faced by the government, namely $D(t) = C_u(t) + C_s(t) - R_t(t)$, where $C_u$ and $C_s$ are the cost of unemployment and CCA subsidy, respectively. While $R_t$ are revenues from firms’ taxes.

However, despite the widespread implementation of bottom-up CCA actions, the compounding direct and indirect adverse effects arising from this extreme flood sequence harm economic growth and the fiscal balance in both the short and long term across all CCA scenarios. This result is in line with the risk layer approaches, where ‘DR’ (disaster risk reduction scenario) and ‘Insurance’ (insurance scenario), either alone or combined (‘DR & Insurance scenario’), can protect the economy from high and medium frequency flooding. Nonetheless, they are insufficient to protect the economy from the devastation of high-return, low-frequency floods such as the one (1000-year return period) that we simulate in 2055. Specifically, the insurance policy has a limit for damages above the 100-year return, and the protective measure is often overwhelmed by a flood of that magnitude. Yet, the magnitude of the negative impact varies considerably across CCA scenarios. Simulation results reveal that the addition of a government subsidy (‘Subsidy & DR & Insurance’ scenario) maintains economic growth and development on a path that is lower but not statistically significant (the significant level is consistent for a two-means t-test and Wilcoxon test) different from the ‘Baseline - No Flood’ scenario (compare purple and black lines in Fig. 2a). Additionally, the role of the subsidy is not statistically significant in the public deficit (compare purple and light blue lines in Fig. 2b).

**Figure 3:**
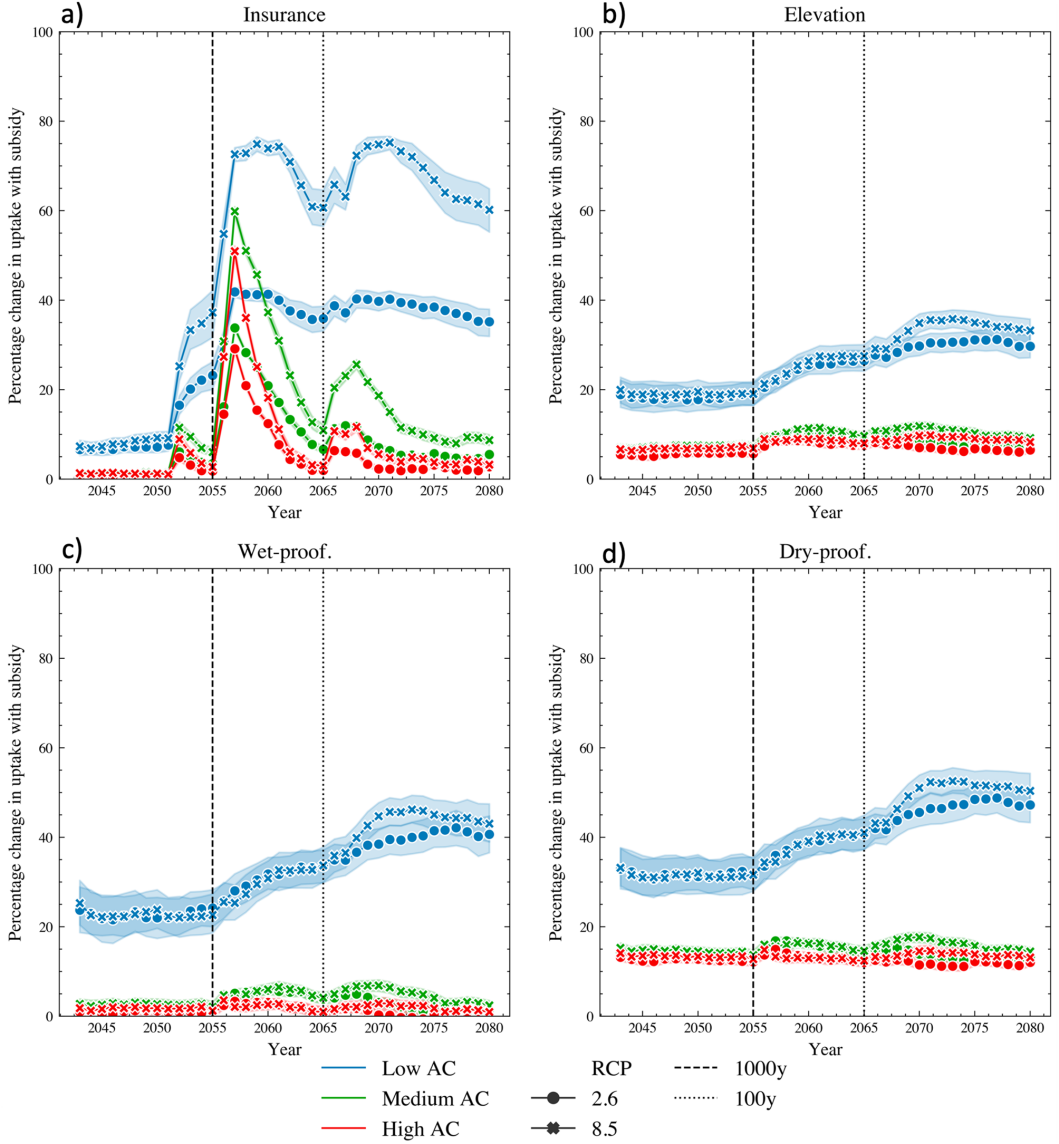
Percentage difference in households CCA actions uptake between ‘Subsidy & DR & Insurance’ and ‘DR & Insurance’ scenarios under RCP 2.6 and RCP 8.5 across households of various adaptive capacities. The reported values are averages across the 100 Monte Carlo runs with the shaded areas denoting the standard deviations. The vertical lines represent the 1:1000 and 1:100 floods.

To understand why the subsidy contributes to the resilient economic development without imposing a heavy burden on public expenses, we must first examine its added value in the regional economy. Specifically, we analyze the difference in adaptation coverage and the related direct and indirect consequences between the scenarios of ‘DR & Insurance’ and ‘Subsidy & DR & Insurance’ across households with different adaptive capacities (ACs). AC is commonly associated with the ability of people and societies to adapt, which is contingent on economic wealth, education, experience, social institutions, and governance^5^. Since the experience, social institutions, and governance are universal for all agents in the CRAB model, we assume that the feasibility of adaptation actions for households depends on their education level and income. Notably, incomes change endogenously in our agent-based model as households change jobs and as the economy develops through technological innovations, but other things being equal, higher educated agents get jobs with higher wages. Since household education grants priority in the CRAB labor market and is highly correlated with income, we anchor AC to the education level (for a more detailed description of the labor market, see “Methods”). Based on this, we distinguish households with Low, Medium, and High ACs. The ACs distribution among the synthetic population of households parameterized with the survey data is 25% High AC, 35% Medium AC, 40% Low AC.

**Figure 4:**
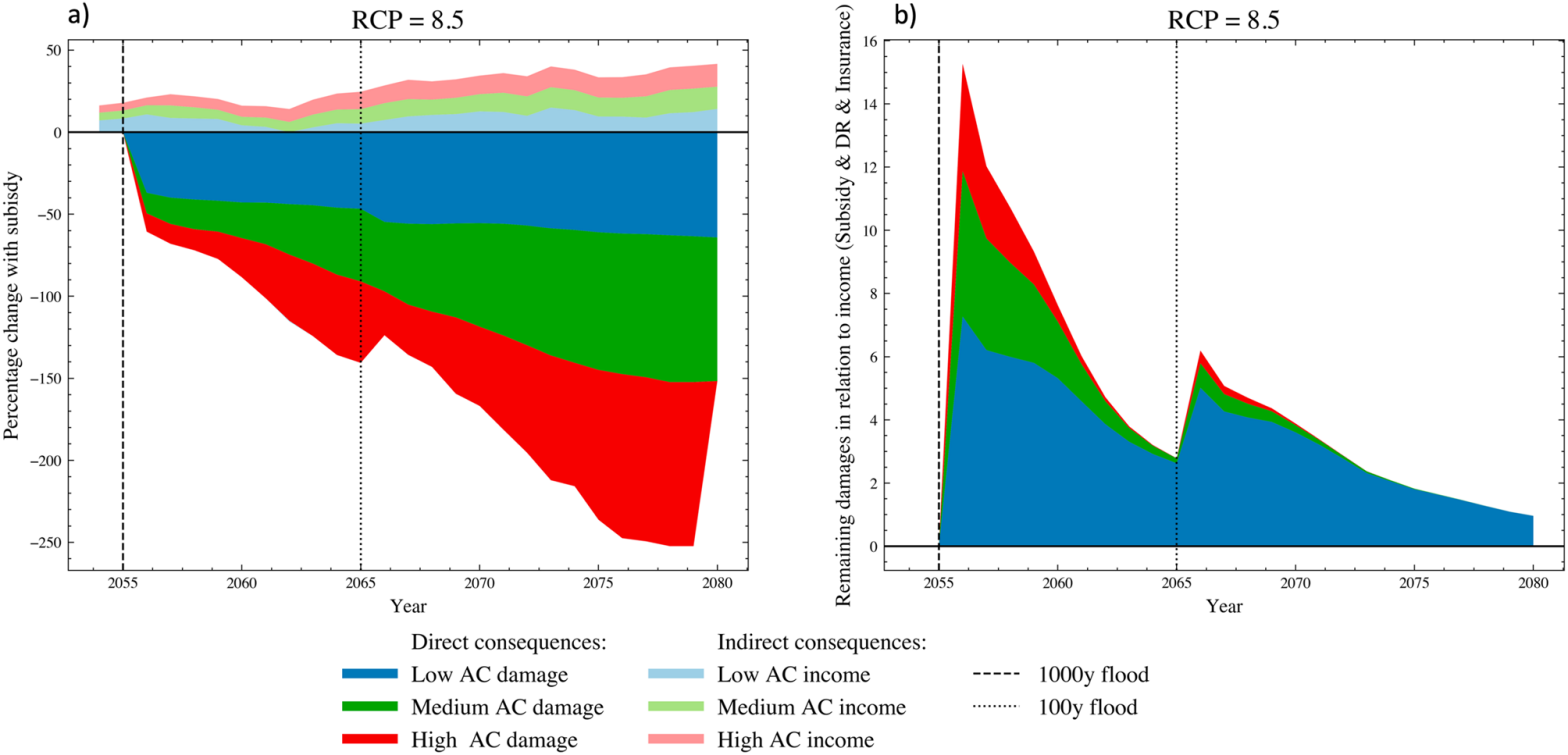
Panel (**a**) displays the percentage difference in direct (repair expenditure) and indirect (changes income) consequences for households with various adaptive capacities between the ‘Subsidy & DR & Insurance’ and ‘DR & Insurance’ scenarios. Panel (**b**)  depicts the remaining damages in relation to the income of households with various adaptive capacities under the ‘Subsidy & DR & Insurance’ scenario. The reported values are the results of stress-testing under the high-end RCP 8.5 scenario, and are averaged across the 100 Monte Carlo runs. The vertical lines represent the 1:1000 and 1:100 floods.

Regarding the individual DR measures (Dry-proofing, Wet-proofing, and Elevation), the subsidy is particularly effective for Low AC households that see a 20-40% increase in all the measures (blue lines in Fig. 3b, c, and d) displaying a willingness to adapt that is more likely constrained by financial resources than the other segment of the population. Conversely, the impact on other ACs is relatively minor, suggesting the presence of ‘soft’ adaptation limits related to individual perceptions, implementation capacity, and social norms^23,37^. Consequently, the uptake of disaster risk reduction measures facilitated by subsidies highly benefits households with low adaptive capacity, which experience a reduction of approximately 50% in direct damages following the first flood (see bright blue in Fig. 4a at the year 2055). Furthermore, simulation results indicate that the subsidy is a crucial factor for insurance when sudden climate changes require households to adapt quickly (see Fig. 3a after the year 2050) and when they need protection in the aftermath of a flood. This is because insurance schemes have a limit beyond which damages are not covered, which in our simulation is set to damages resulting from a 1:100 years flood. As a result, insurance does not cover a substantial portion of the damages caused by the 1000-year flood that occurs in the year 2055. Under such circumstances, households prioritize their resources towards repair costs and do not renew their insurance subscriptions unless the government subsidizes them (see Fig. 3a after the year 2055). Notably, with the subsidy, they remain fully covered, and households of all AC levels recover faster, resulting in an increasing difference in remaining damages over time compared with the scenario with no subsidy (see bright color areas in Fig. 4a after the year 2065).

Overall, the implementation of top-down and bottom-up CCA actions reduces damages for all households, yet residual damages, particularly from extreme 1000-year floods, remain significant as individual measures cannot guarantee full protection (see Fig. 4b at the year 2055). Particularly, households with lower adaptive capacity still bear higher residual damages and require more time to recover. The issue of recovery is compounded when these households are subjected to consecutive shocks, challenging their resilience and amplifying the extent of damages. The ability to recover is also influenced by indirect damages as captured by the CRAB model through labor market interactions. Households with low adaptive capacity are more prone to job losses and experience difficulties finding new employment, thus losing their steady income post-flooding. Consequently, they increasingly depend on unemployment subsidies and lack adequate resources for property repair and flood recovery. In the CRAB model, households aim to repair their property as swiftly as possible by saving each additional income above the unemployment subsidy, which we assume is the minimum level to satisfy basic needs. Nevertheless, it is noteworthy that the incorporation of CCA actions, specifically a mix of top-down and bottom-up measures, creates indirect advantages for the wider economy. These economic benefits in turn positively affect household income levels, reducing the compounding effect of recurrent risks (shaded regions in Fig. 4a). The exploration of how these indirect dynamics unfold and their role in mitigating the compounding effects of repetitive hazards forms the focus of the following section.

### Top-down and bottom-up CCA strategies offer the best chance to build a climate-resilient regional economy against compounding hazards

This section explores the potential for combining a top-down subsidy with bottom-up CCA actions to enhance economic resilience in the CRAB regional economy in a cost-effective manner. The CRAB model examines the direct consequences of floods, such as the destruction of properties and machinery, as well as the indirect consequences and feedback loops that arise from the interactions among economic agents following the shock.

**Figure 5:**
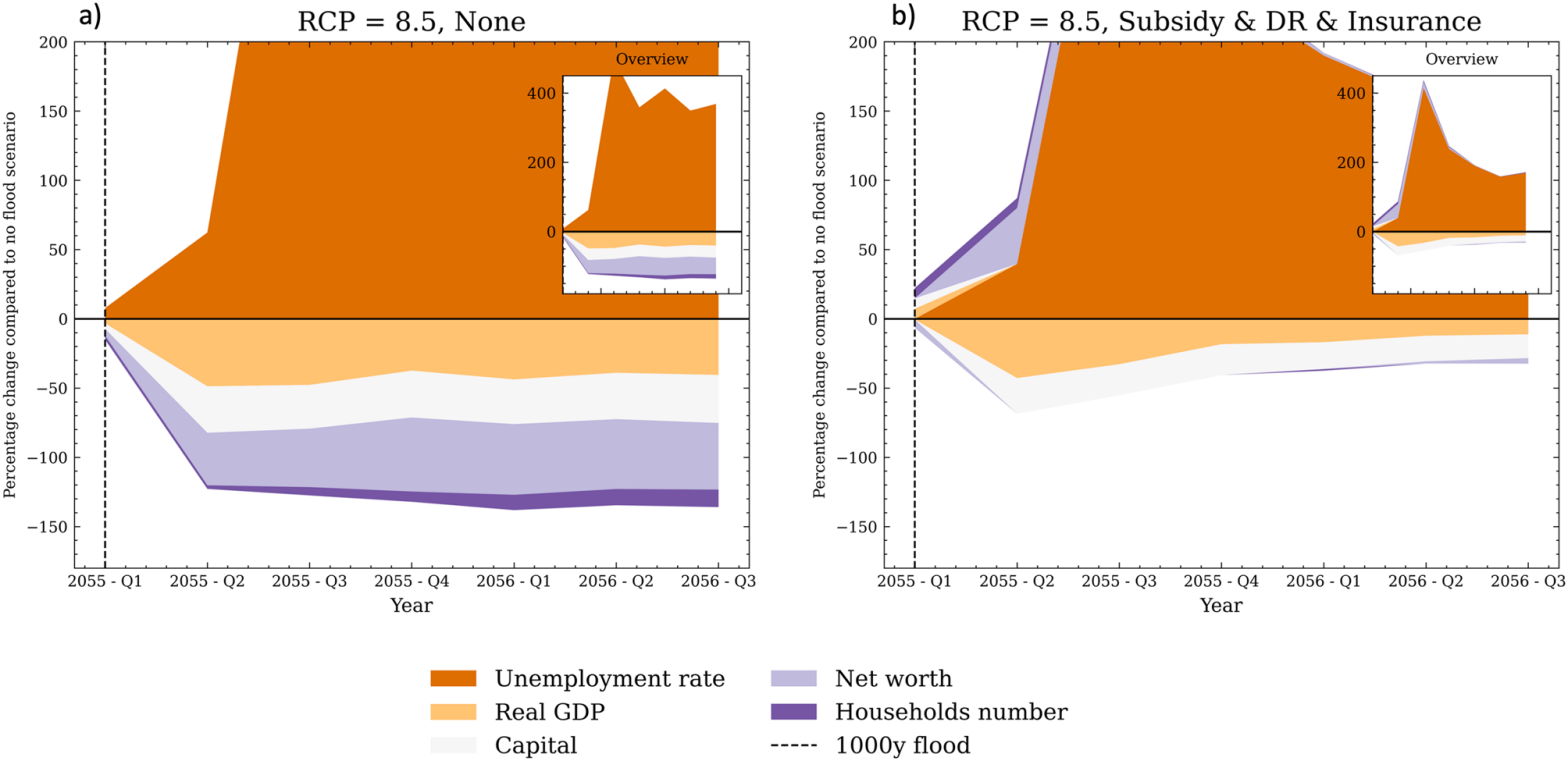
Panel (**a**) shows the percentage difference of the short-term direct and indirect consequences in the CRAB regional economy when no adaptation action is taken (‘None’) compared to the ‘Baseline - No flood’ scenario. Panel (**b**) makes the same comparison with the ‘Subsidy & DR & Insurance’ scenario. The reported values are the results of stress-testing under the high-end RCP 8.5 scenario, and  are averaged across the 100 Monte Carlo runs. The vertical lines represent the 1:1000 and 1:100 floods.

**Figure 6:**
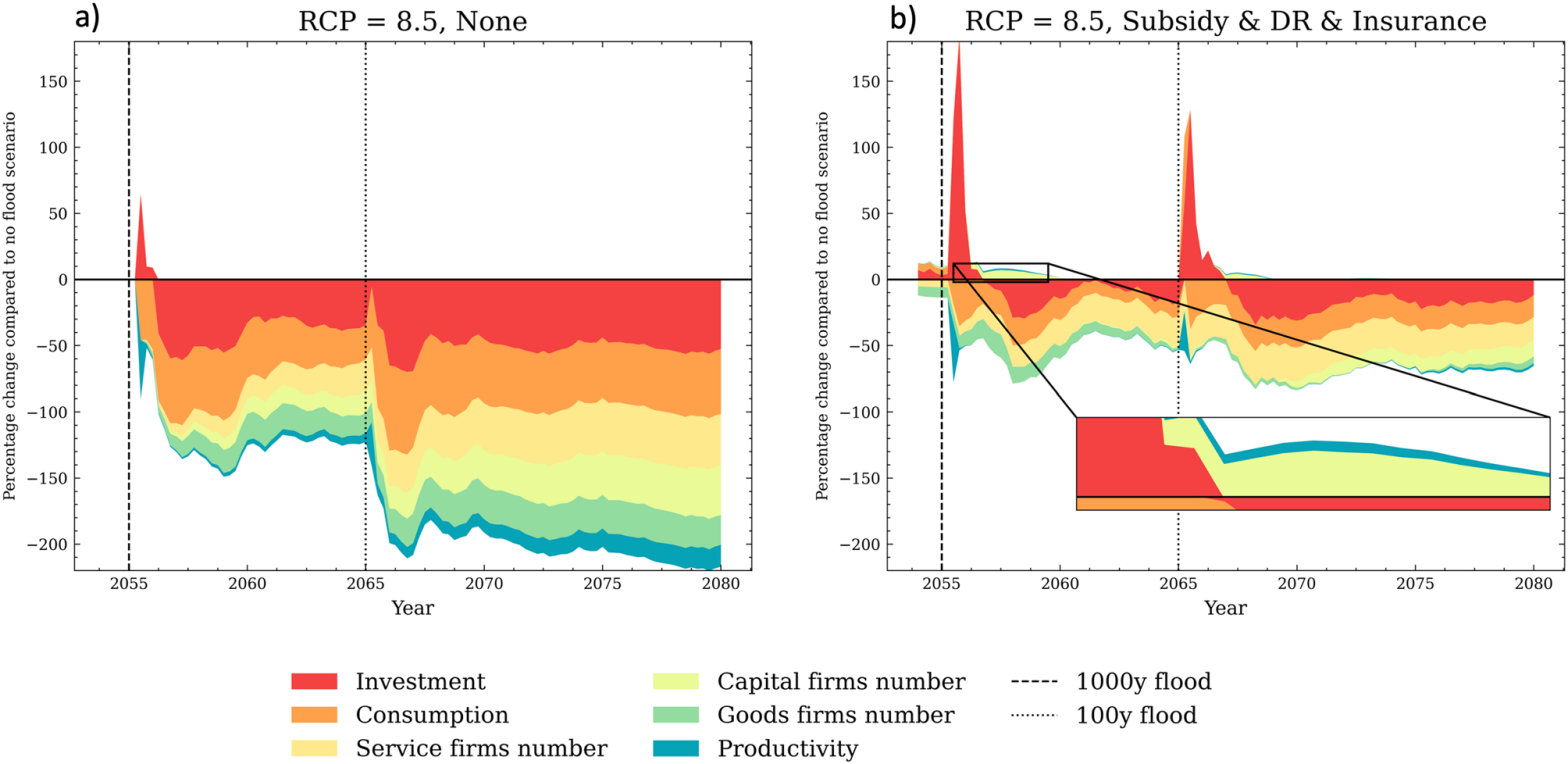
Panel (**a**) shows the percentage difference of the long-term direct and indirect consequences in the CRAB regional economy when no adaptation action is taken (‘None’) compared to the ‘Baseline - No flood’ scenario. Panel (**b**) makes the same comparison with the ‘Subsidy & DR & Insurance’ scenario. The reported values are the results of stress-testing under the high-end RCP 8.5 scenario, and are averaged across the 100 Monte Carlo runs. The vertical lines represent the 1:1000 and 1:100 floods.

One key interaction that unfolds in the aftermath of floods is the destruction of firms’ machinery, which creates a need for liquid resources to rebuy capital. However, not all firms have sufficient resources to replace their capital stock, resulting in downscaled production and layoffs (see brown areas in Fig. 5a). This, in turn, leads to an increase in the unemployment rate and a decline in income per capita, making the region less attractive economically and leading to household out-migration (see purple areas in Fig. 5a). In the long-term, the out-migration of households leads to lower consumption and reduced internal demand for goods and services (see orange area in Fig. 6). Additionally, damaged households allocate more resources to repairing their properties, resulting in increased spending on goods and less on services. Firms in the CRAB model are boundedly rational and have imperfect information, making steady demand beneficial for their production plans and hiring process. Abrupt changes in consumption patterns due to floods make planning harder for firms and increase the likelihood of an economic downturn. All of these factors combined make the region less profitable for firms, resulting in a lower entry rate compared to the baseline scenario. Despite the initial surge of investment to replace destroyed capital (see read area peak at the year 2055 in Fig. 6a), the lack of profitability and resources hampers long-term investment by firms, thereby limiting technological development and productivity growth in this regional economy, hence triggering the compounding of the longer-term losses. Simulation results suggest that the cost of inaction in response to these extreme events could weaken or even break the agglomeration that drives the high economic growth and development of regions with coastal megacities.

Importantly, the combination of top-down and bottom-up CCA action can help mitigate these negative effects. In particular, protective measures such as disaster risk reduction can decrease repair costs and keep expenditures stable, thereby aiding firms’ production plans. Timely paid insurance also plays a critical role in providing firms with enough liquidity to replace their damaged machinery, minimizing business interruption, and output losses. This has a positive impact on the unemployment rate, which, in turn, softens the negative effect on income per capita and discourages household out-migration from the region (see brown and purple areas in Fig. 5b). Furthermore, households and firms with more resources to speed up the recovery in the post-flood period results in higher investment, minimizing long-term losses of profitability, productivity and technological innovation. The ‘forced’ investment and capital replacement that firms undertake following climate-induced shocks can even trigger a creative destruction process during the post-flood period (the so-called ‘productivity effect’ or ‘build back better’^38^ see zoomed area in Fig. 6b). However, it is important to note that this effect fades over time as residuals investments are still lower than the ‘Baseline - No flood' scenario.

A top-down subsidy plays a crucial role in maintaining the protection of economic agents against the compounding effect of the second flood while recovering from the first and increasing coverage for more vulnerable individuals and businesses. This approach is cost-effective for the government, as it allows firms to maintain business activity and not get bankrupt, indirectly increasing government revenues via taxes. Another co-benefit of this multi-scale CCA is that fewer workers are laid off, reducing government expenditure on unemployment subsidies. The initial government expenditure works to reduce the compounding indirect consequences of consecutive floods, eventually benefiting the government budget.

The role played by the top-down government subsidy highlights the need for coordinated CCA actions across scales to build climate-resilient societies and limit compounding risks and spillovers between anthropogenic and natural processes. The integration of bottom-up and top-down approaches enables a more comprehensive and effective strategy to enhance economic resilience to climate-induced shocks, protecting vulnerable individuals and businesses and reducing the negative impact on the government budget.

## Discussion

The primary objective of this study was to investigate the compound risks arising from the dynamics interactions between endogenous economic growth, multi-scale adaptation, and the direct and indirect damages caused by repetitive climate-induced shocks. This exploration aims to bridge the gap in understanding the compounded effects of recurring hazards in the context of climate-induced hazards. We do that by employing an innovative combination of an evolutionary economic ABM, multi-layer risk management strategies, and rich survey data to analyze the role of different CCA strategies in a complex evolving regional economy. We demonstrated how it is possible to jointly include direct flood damages, which dynamically vary depending on agents’ protective CCA actions, indirect damages, and recovery, which evolve following the interactions among different stakeholders in the aftermath of the disaster. The latter are aspects that are largely omitted in the flood-ABMs literature. To inform households’ socioeconomic and behavioral attributes, we utilized rich behavioral data from the Shanghai area and loosely calibrated the regional economy to resemble an archetype of an agglomerating coastal resource-rich megacity with characteristics akin to that of Shanghai.

Our analysis focuses on ripple effects generated by the interactions of firms and households in a regional economy that experience extreme climate-induced flooding. Specifically, our work intends to shed light on possible indirect costs and benefits of different CCA strategies and explore possible synergies that generate positive externalities on socio-economic resilience cost-effectively. Using 600 runs of the agent-based model, with each experiment consisting of 100 Monte Carlo runs, we quantify the implications of private CCA measures alone and in  combination with a public subsidy on the economic and fiscal resilience of a fast-growing coastal regional economy. This work contributes to a new generation of computational models encompassing risk and applying a resilience perspective to complex adaptive systems^20,39^.

In addressing our first research question, we explored the compounding impacts of two consecutive floods on the regional economic growth and fiscal budget under different CCA adaption strategies. We found that only with CCA actions undertaken at all levels of society, both top-down and bottom-up, it is possible to maintain a climate-resilient society with long-term economic growth and development comparable to a stable climate. These findings highlight the indirect fiscal benefits of a government subsidy for private CCA by maintaining tax revenues from firms’ profits and reducing costs related to unemployment subsidies. These results align with previous research on preventive public expenditure, which can improve economic resilience, hasten recovery, and mitigate the impact of shocks, with indirect benefits that can outweigh initial costs^40^. Additionally, the simulation results reinforce the significance of incorporating CCA strategies across scales that take into account multiple stakeholders beyond direct damages, which has been extensively discussed in previous literature^24^ and highlight how inadequate actions result in feedback loops that do not reduce existing flood risks. Furthermore, the conclusions emphasize the need to revise current adaptation measures that focus primarily on singular flood events. The escalating frequency and intensity of such incidents, driven by climate change, underscore the necessity to address the compounded risks arising through both direct and indirect damage channels. This approach ensures resilience not just to one-off shocks, but also to their repeated and intensifying occurrences that could otherwise trigger cascading socio-economic repercussions^25,41^.

To answer the second research question, we focused on assessing the effectiveness of the subsidy in promoting adaptation measures and reducing both direct and indirect damages for individuals with heterogenous adaptive capacity. Our results indicate that the subsidy is particularly effective in increasing the uptake of protective measures among the most vulnerable Low AC individuals who are mainly constrained by financial reasons and lack of resources. At the same time, the effect of subsidies for Medium and High AC is negligible, pointing to the presence of other adaptation constraints rather than financial. Additionally, the model results demonstrate that the subsidy is helpful in sustaining insurance uptake when climate change is fast, and individuals prioritize spare resources for repair costs after a flood, resulting in compounding vulnerability to further consecutive  shocks. Our analysis also highlights that while the subsidy is essential in situations of rapid climate change and in the aftermath of floods, it may not be adequate in overcoming other non-financial soft limits that can generate an ‘adaptation deficit’, leading to adaptation levels remaining below optimal levels^4,23^. These findings support existing research on the role of subsidies in overcoming financial constraints and emphasize the need for targeted interventions that can address other soft limits of adaptation^37^. Furthermore, the CRAB modeling framework reveals that by decreasing the repair cost needed after a flood and speeding up the recovery of residual damages, particularly for the 100-year flood, the subsidy generates indirect benefits to the regional economy. This generates positive feedback to households, resulting in fewer income losses in the long run. It is crucial to note that despite these benefits, more vulnerable households are still the category that is most affected by floods, primarily due to difficulties experienced in the reshuffling of the labor market. Our simulation results reinforce previous literature findings that poor people are marginally more impacted by disasters and struggle to recover due to job and income losses^42,43^. Moreover, they highlight the critical but not sufficient role played by bottom-up protective actions in reducing flood risk^5^. Overall, our study underscores the importance of implementing targeted and multifaceted approaches for building resilience to climate change, particularly among vulnerable populations. This perspective resonates with a growing body of academic work that highlights the need of convergence between state-driven economic growth and strategies for climate resilience and adaptation^44^.

To address our third research question, we compared the evolution of several variables, both short- and long-term, between the ‘None’ and ‘Subsidy & DR & Insurance’ scenarios. Specifically, we examined regional dynamics and explored the cost of inaction, as well as how such costs change in the case of top-down and bottom-up CCA strategies. Our findings demonstrate that the additional resources provided by insurance enable firms to minimize business interruption, leading to a positive impact on output and the unemployment rate. This contributes to maintaining the economic attractiveness of the region and minimizes outmigration in the aftermath of a flood. Moreover, these additional resources increase the amount of post-flood investment, opening up the possibility of a window of opportunity, in which destroyed machinery is replaced with new and more productive equipment. Our results are consistent with previous literature highlighting the critical role of business interruption in reducing indirect damages and enhancing socio-economic resilience^45^. These results have significant policy implications, demonstrating how the cost of inaction can reach a tipping point that breaks agglomeration forces, resulting in high opportunity costs for potential future development. Conversely, providing resources that expedite recovery after a flood has invaluable indirect benefits for the overall region. Additionally, our results emphasize that a ‘build back better’ pathway is possible but requires sustained investment to transform risk into opportunities. Our framework advances previous flood-ABMs literature by being the first model that includes both firms’ and households’ CCA actions in a complex socio-economic environment calibrated with rich behavioral data^20^.

The CRAB model can be extended in several ways. First, the model would benefit from more extensive Monte Carlo simulations. Specifically, a full stochastic approach to extreme flooding would measure the full spectrum of risks associated with rare but impactful events. While this would pose significant computational challenges, given the rarity of these events, it would greatly enhance our insights into the multitude of ways in which consecutive extreme events could impact the socio-economic system. Second, a richer calibration on the economic side, such as including empirically calibrated NAICS sectors, would lead to more precise estimation of economic impacts. Furthermore, governments and firms are known to take protective CCA actions, such as dykes and levees, to reduce the adverse impacts of hazards. Hence, protective actions across multiple scales and stakeholders could be jointly considered to analyze both limits and opportunities that regions have for development despite adversities. In addition, in our exploration of adaptation strategies, it’s pertinent to consider the evolution of the insurance market. A more sophisticated insurance paradigm, where premiums are discounted for agents actively engaging in protective measures, can serve as a significant incentive. This not only fosters proactive risk management but also mirrors the dynamic interplay between market-driven mechanisms and adaptation behaviors. Another important avenue for future research would be the introduction of more detailed migration patterns coupled with land-use dynamics, allowing the differentiation between urban, peri-urban, and rural areas. Regardless of these limitations, the CRAB model provides a general understanding of the importance of a comprehensive framework that includes both firms and households and how their interactions and cumulative individual actions shape regional climate-induced damage and resilience. Importantly, the model offers new ways forward to tackle not only the direct but also indirect risk in an explicit way and therefore offers new ways forward how to transform emerging risk challenges into long-term opportunities.

## Methods

### The Model

We introduce a novel version of the Climate-economy Regional Agent-Based (CRAB) model^22,23^ to account for indirect damages caused by floods. This model combines households and firms in a regional economy, where they interact through market institutions, migration, climate, and technological learning. Such interactions enable the mapping of feedback loops and cascading effects generated by flood shocks. Additionally, we incorporate a risk layering strategy consisting of bottom-up and top-down adaptation strategies that range from individual risk reduction to insurance. By leveraging rich behavioral data, we parameterize the model to represent an archetype of a coastal resource-rich megacity. The CRAB model builds on the evolutionary economic engine of the ‘Keynes + Schumpeter’^46–48^ and ‘Dystopian Schumpeter meeting Keynes’^16,49^ models. This novel version features a three-sector regional economy with four classes of heterogeneous, boundedly-rational agents that dynamically interact in decentralized capital, labor, and good/service markets with households. The number of agents varies depending on the migration flow for households, while firms follow independent entry and exit processes (for a detailed description, see SI; “Model Complements”). The region is exposed to flooding with varying return periods, whose severity increases with climate change over time. Floods impact agents at the microeconomic level, resulting in the destruction of household properties, firm inventories, and machines. Households and firms residing in flood-prone areas can take multiple adaptation actions to protect themselves. Technological learning and economic growth are driven by a creative destruction ‘Schumpeterian’ process.

### Firms

In this novel version, all the macro sectors in the regional economy require both capital and labor as production inputs. In line with the ‘K+S’ tradition, the capital-good sector invests in R &D and tries to discover more productive technologies. The latter generates a ‘Schumpeterian’ creative (innovative) destruction process, which is the engine of endogenous economic growth. Machines are then advertised through “brochures” to possible customers, which in this version are all the other firms. Once orders are received, capital-good firms estimate their expected demand and required machines to produce it. If the current stock of machines is insufficient to satisfy the desired production, additional ones are ordered from other capital-good firms. We assume that capital-good firms cannot self-produce the capital they need for themselves, but they need to order it from other capital-good firms. Once the capital market closes, capital-good firms enter the labor market, trying to hire the optimal number of workers for their feasible production. Finally, they combine capital and labor to produce machineries that will be delivered in the next step. In a similar manner, consumption-good/-service firms combine labor and machines to produce a homogenous good/service for consumption. The latter two sectors follow the same decision-making process by using adaptive heuristic demand expectations and fixed capital-output ratios. Importantly, if capital stock is insufficient to satisfy the desired production, new machines are ordered comparing the ‘brochures’ they are aware of. In addition, all the firms can replace current machines by using a pay-back rule. Notably, the three macro sectors’ capital requirements are different as they have different capital-output ratios. Following empirical evidence^33^, we parametrize the capital sector to be the less capital intensive, followed by goods, and finally, services, which is the most capital intensive. In addition, firms living in flood-prone areas can buy insurance that refunds destroyed machineries and inventories in case of a flood.

### Households

In the CRAB model, households are characterized by multiple socio-economic and behavioral factors, calibrated with survey data. Socio-economic characteristics include property values, education, and initial savings. Households’ income evolves through interactions with firms in the labor market, where more educated households have better access to job opportunities. Unemployed households receive a subsidy from the government.

In each time step, households spend all their income unless they plan to save for a protective action or insurance premium. Household intention to undertake a protective action depends on behavioral characteristics, including social interactions in a random social network. The Protection Motivation Theory (PMT) is used to estimate households’ protective actions against flooding^50^.

Weights of each PMT attribute are estimated with a logit regression. Each household calculates its intention to undertake a climate change adaptation action using the PMT attributes and their weights. The intention-action gap is accounted for by multiplying the intention probability by a factor lower than one. Household action probability is determined by comparing it to a random number.

Households with a positive action probability save all income above the minimum wage until they have enough resources to implement the protective measure. Protective measures affect monetary damages in case of a flood, with the amount of damages depending on the household property value and the damage coefficient. Damaged households follow the same saving mechanism for protective actions.

The value of household property is indexed to the region’s average wage, increasing over time with technological learning and economic growth. The cost of the action and repair cost is added to the aggregate demand of the consumption-good sector.

### Markets

In an economy, households and firms interact through socio-economic institutions, such as markets for capital, labor, and goods/services. In the capital market, capital-good firms send brochures to their existing and new potential customers, which contain the price and productivity of their machines. Firms seeking to buy new machines compare the brochures and select the supplier offering the best price-quality ratio. In the labor market, firms assess their labor demand and then post available vacancies or fire surplus workers. Unemployed households, sorted by education level, choose available vacancies and select the one offering the highest wage, resulting in a positive correlation between income and education.

Salaries earned by households are spent on goods and services, including insurance, which is a subcategory of the service market. The insurer calculates the expected annual damages for an agent by integrating the damages caused by a flood with the flood probability at the entry point. For households, damages are calculated by multiplying the house value by the damages coefficient, while for firms, damages are calculated by multiplying the fraction of capital stock and inventories that would be destroyed by the flood with their current market prices.

The insurer determines the market price of insurance cost for an agent at time *t* by adding a fixed markup to the expected annual damages. All agents are assumed to be risk-averse, so they will subscribe to the insurance if they have the resources. The model’s underlying assumption of agents being risk-averse stems from empirical evidence indicating heightened risk aversion following natural disasters, particularly at a localized level^51^. Importantly, insurance expenditures contribute to the aggregate demand of the service sectors. In the event of a flood, the insurer claims are shared among the service firms proportionally to their market share. Protective measures and repair costs incurred are added to the aggregate demand of the goods sector.

The local demand is defined by the aggregate household expenditure in the goods and service markets, which is summed with export demand and assigned to firms based on their market share. Firms’ market share evolves through quasi-replicator dynamics, which depend on their competitiveness, calculated according to their prices and unfilled demand.

### Entry and exit process

In order to integrate the agglomeration process into the regional economy, households’ and firms’ entry and exit processes are independent in this novel version of the CRAB model. Specifically, a migration process linked to regional economic indicators regulates the number of households. In tune with empirical evidence, we use the difference in income per capita and the unemployment rate as reference variables^52^. In a nutshell, an economy with growing income per capita and a low unemployment rate attracts new entrants sampled from the synthetic population pool and added to the incumbents. Conversely, a stagnant economy will push households to leave. Households also affect the creation of new firms from the bottom up. In particular, an employed household decides to create its own firm if the profits of its current employer exceed a certain threshold for a number of consecutive periods. Firms with quasi-zero market share and lack of resources are removed.

### Supplementary Information

Supplementary Information 1.

## Data Availability

The datasets used and/or analyzed during the current study available from the corresponding author on reasonable request.
