# Supplementary material for: Economic implications of autonomous adaptation of firms and households in a resource-rich coastal city
Source: Sci Rep. 2023 Nov 21;13:20348. doi: 10.1038/s41598-023-46318-2 (PMC10663627; doi:10.1038/s41598-023-46318-2)
Supplement: Supplementary file 1 — Supplementary Information 1. [file 41598_2023_46318_MOESM1_ESM.pdf]

Economic implications of autonomous adaptation of firms and households in a resource-rich coastal city

Alessandro Taberna[1,2 *], **Tatiana Filatova**[1,*], **Stefan Hochrainer-Stigler**[2], **Igor Nikolic**[1], and **Brayton Noll**[1]

[1]Delft University of Technology; Faculty of Technology, Policy and Management; Department of Multi Actor Systems; Jaffalaan 5, 2628BX, Delft, The Netherlands
[2]International Institute for Applied Systems Analysis; Schlossplatz 1, 2361 Laxenburg, Austria
[*]a.taberna@tudelft.nl, t.filatova@tudelf.nl

ABSTRACT

Climate change intensifies the likelihood of extreme flood events worldwide, amplifying the potential for compound flooding. This evolving scenario represents an escalating risk, emphasizing the urgent need for comprehensive climate change adaptation strategies across society. Vital to effective response are models that evaluate damages, costs, and benefits of adaptation strategies, encompassing non-linearities and feedback between anthropogenic and natural systems. While flood risk modeling has progressed, limitations endure, including inadequate stakeholder representation and indirect risks such as business interruption and diminished tax revenues. To address these gaps, we propose an innovative version of the *Climate-economy Regional Agent-Based* model that integrates a dynamic, rapidly expanding agglomeration economy populated by interacting households and firms with extreme flood events. Through this approach, feedback loops and cascading effects generated by flood shocks are delineated within a socio-economic system of boundedly-rational agents. By leveraging extensive behavioral data, our model incorporates a risk layering strategy encompassing bottom-up and top-down adaptation, spanning individual risk reduction to insurance. Calibrated to resemble a research-rich coastal megacity in China, our model demonstrates how synergistic adaptation actions at all levels effectively combat the mounting climate threat. Crucially, the integration of localized risk management with top-down approaches offers explicit avenues to address both direct and indirect risks, providing significant insights for constructing climate-resilient societies.

Model complements

The *Climate-economy Regional Agent-Based* (CRAB) model builds upon the evolutionary economic engine of the well-validated "Keynes + Schumpeter"[1,2] and the "Dystopian Schumpeter meeting Keynes"[3,4] models. The regional economy of the model consists of $F1$ heterogeneous capital-good firms (denoted with the subscript i), $F2$ consumption-good firms (denoted with the subscript j), $F3$ consumption-service firms (denoted with the subscript l) and H households (denoted with the subscript h) supplying work and consuming the income they earn. When a decision process is identical for all firms (e.g. migration), we employ the subscript f. In addition, to generally refer to consumption firms (both service and good), we use the subscript s.

Households

The CRAB model includes behaviorally rich and heterogenous household agents, whose attributes are summarized in Table S2. Notably, households are embedded into a random network, which takes the form of a dynamic Erdos-Reny random graph[5]. Each node of the graph contains a household and it has a number of edges that connects it to the initial number (n) of nearest neighbors. At initialization, we set $n = 7$ to align the initial number of household connections in the survey data (see Table S2). However, the number of connections evolves over time in the simulation, for example due to migration. Specifically, when a new household enters the regional economy, the existing network adds a new node and connects it to n existing nodes, which are randomly sampled. Furthermore, when a household leaves the region, its node is removed with all its edges. Hence, together with the network, the number of household connections evolves endogenously and it is contingent on the migration flow.

Migration

The migration flow of households depends on the regional economic performance. Empirical evidence shows that expected income and employment opportunities are the main factors driving inter-regional household migration[6]. Hence, we use these two factors in regulating the inflow and outflow of households (b^h) at time t in the region:

$$b^h(t) = H(t)[(1-o)IA(t) + o\pi(t)], \quad with \begin{cases} b \leq 0 & \text{if } U(t) < U_{max}(t) \\ b \geq 0 & \text{if } U(t) > U_{min}(t) \end{cases} \tag{1}$$

where $H(t)$ is the existing number of incumbents households, $IA(t)$ is the "income attractiveness" of the region, $0 \leq o \leq 1$ is a mix balance parameter, and $\pi(t)$ is a random draw from a uniform distribution on the fixed support $[\underline{x_2}, \overline{x_2}]$. $U_{[min,max]}$ are the minimum and maximum unemployment rates that make people come and leave, respectively. The number of entrants stochastically depends on the number of incumbents (recalling a spin-off process of the former from the latter), with the income conditions influencing the decision of potential entrants[7]. The "income attractiveness" $IA(t)$ is defined as:

$$IA(t) = Ipc(t) - Ipc(t-1), \text{ bounded to } [\underline{x_2}, \overline{x_2}], \tag{2}$$

with Ipc at time t is given by the sum of all individual income In_h (defined below see Eq.13) and wealth, divided by households population as:

$$Ipc(t) = log(\sum_{h=1}^{H} In_h(t) + \sum_{h=1}^{H} W_h(t)) - logH(t) \tag{3}$$

Where $W_h(t)$ is the value of household h property (for its evolution, see Eq.5) at the net of monetary damages (D_h, for more information about damages calculation see Eq.6) to be repaired at time t:

$$W_h(t) = Hv_h(t) - D_h(t) \tag{4}$$

If positive ($b > 0$), new households are added, sampled randomly from the synthetic population pool (for more information about the creation of the synthetic population, see Subsection "Model calibration"). If negative ($b < 0$), random households are selected and removed from the simulation.

Damages and adaptation

We assume that every household resident in the region owns a property, whose value (Hv_h) is indexed to average regional salaries[1]:

$$Hv_h(t) = Hv_h(t-1)\frac{\overline{W}(t) - \overline{W}(t-1)}{\overline{W}(t-1)}, \tag{5}$$

with $\overline{W}$ being the average salary of the regional economy.

Importantly, household properties can be damaged by flooding. Flood property damages ($D(t)$) at time t are calculated by multiplying household h damage coefficient (Dc_h) by its house value:

$$D_h(t) = Hv_h(t) * Dc_h(t). \tag{6}$$

The damage coefficient ($Dc \in [0,1]$) is obtained from overlying flood depth hitting the property (for more information about the distribution of flood depths, see Subsection "Floods") as a function of the specific residential depth-damage curve[9]. Notably, households can decrease their damage coefficient by undertaking adaptation measures to their property. Our survey elicits households' intentions to invest in 7 structural CCA measures, which we group into three classes here: *Dry-proofing*, *Wet-proofing*, *Elevation*[10,11]. The data on the effectiveness of these various CCA is scattered, so here we take average values previously employed in the literature. The *Dry-proofing* and *Wet-proofing* measures decrease households damage coefficient Dc with a fixed amount $\alpha_{Dry,Wet}$, while *Elevation* diminishes flood depth (d) of α_{Elev}:

$$Dc_h(t) = (1 - \alpha_{Dry} - \alpha_{Wet}) f(d - \alpha_{Elev})(t), \quad with \begin{cases} 0 < \alpha_{Dry,Wet} < 1 & \text{if Dry, Wet is implemented} \\ \alpha_{Dry} = 0 & \text{if d > fe} \\ \alpha_{Elev} > 0 & \text{if Elev is implement} \\ \alpha_{Dry,Wet,Elev} = 0, & \text{if measure is not implemented} \end{cases}, \tag{7}$$

[1]The assumption is reasonable as wage levels are strongly correlated with house price[8].

With fe being the maximum flood height that *Dry-proofing* measures can stand (i.e. not over-topping flood barriers, for an overview of the relevant parameters for the protective measures see Table S5).

Households are boundedly-rational and employ an extended version of Protection Motivation Theory (PMT)[12] to decide upon protective actions. PMT is currently the most widely used psychological theory to study individual CCA decisions. Here we employ its extended version where an individual intention to take CCA actions is driven by own threat appraisal (perceived probability; perceived damage; worry) and coping appraisal (response-efficacy; self-efficacy; perceived costs) as well as the influence of social norms, previous experience with floods and with CCA[13]. Each time step in CRAB, households at risk of flooding calculate the adaptation *intention* probability $p^{int}_{cca_m} \in [0,1]$ for each *cca* measure (m) by multiplying individual behavioral attributes at time t by their effect size, obtained from a Logit regression (for a detailed description of behavioral attributes see Table S2, while for effect sizes see Table S3).

$$p^{int}_{cca_m,h}(t) = \frac{1}{1+e^{\beta_0+\sum_{a=1}^{15}\beta_a X_a(t)}} \tag{8}$$

Here, β_a and $X_a(t)$ are the effect sizes and attributes, respectively. Notably, some attributes change over time. When contemplating any specific action cca_m from $cca = \{Dry, Wet, Elev\}$, the historical adaptation measures for all other actions are represented by:

$$UG_n = \begin{cases} 1 & \text{if } cca_n \text{ adaptation measure undertaken previously, } n \neq m \\ 0 & \text{otherwise} \end{cases} \tag{9}$$

for all n in CCA where $n \neq m$. This denotes whether household h has implemented other measures in the past while considering a particular measure cca_m. The overall regional adaptation level affects households through social interaction, with *Soc. network* that evolves endogenously as:

$$Soc.\ network_{cca_m,h}(t) = \sum_{h=0}^{H^{net,h}_{cca_m}} 1, \tag{10}$$

with $H^{net,h}_{cca_m}$ being the set of households connected to household h and that have already implemented the *cca* under consideration.

Empirical evidence shows that there is a consistent gap between intentions and the actual behavior[10]. Hence, relying on this empirical data, in CRAB we assume that the probability to act p^{act} at time t as:

$$p^{act}_{cca_m,h}(t) = \Phi p^{int}_{cca_m,h}(t), \tag{11}$$

with $\Phi < 1$ that reflects the degree of the intention-behavior gap.

Finally, households with $p^{act}_{cca_m} > 0$ draws from a Bernoulli distribution - in the similar fashion as for migration and technological learning (see Subsection "Migration" and Subsection "Capital-good sector and technological learning") - to determine whether adaptation occurs:

$$\theta_h^{cca_m}(t) = p^{act}_{cca_m,h}(t), \quad with \quad p^{act}_{cca_m,h}(t) \in (0,1). \tag{12}$$

If the draw is successful, the household starts to save a fraction of its personal income until she can cover the implementation cost ($cost_{cca_m}$).

Households get their income (In_h) from labor when employed, while unemployed households get a subsidy from the government (w^u):

$$In_h(t) = \begin{cases} w_h(t), & \text{if employed} \\ w^u(t), & \text{if unemployed} \end{cases}. \tag{13}$$

Each time step, unemployed households sort in ascending order by their education level, visit the labor market, and select a sub-sample of available vacancies (if any), choosing the one with the highest wage (for more information about the labor market, see Subsection "Labor"). Having priority in the queue, more educated households are more likely to get better-paid job opportunities than their less-educated peers. Households consume all their income unless two conditions happen, namely, they want to implement protective CCA actions to their house, or they have to undertake repair damages due to floods. When these conditions happen, we assume households want to minimize the time to acquire the necessary resource and save all their

extra income above the unemployment subsidy (which we assume is the minimum to satisfy basic needs). Thus, household consumption (c_h) at time (t) is:

$$c_h(t) = In_h(t) - Sav_h(t), \tag{14}$$

where $Sav(t)$ are the savings of household h at time t and are defined as:

$$Sav_h(t) = \begin{cases} In_h(t) - w^u(t), & \text{if } D_h(t) > 0 \quad or \quad cost_{cca_m}(t) > 0 \\ 0, \text{otherwise} \end{cases}, \tag{15}$$

Each time step, savings go first into residual damages to be repaired, which hence decrease over time accordingly to:

$$D_h(t) = D_h(t-1) - Sav_h(t). \tag{16}$$

Alternatively, households without without damages to be repaired ($D(t) = 0$) and with a planned cca measure, will gradually cover its cost:

$$cost_{h,cca_m}(t) = cost_{h,cca_m}(t-1) - Sav_h(t). \tag{17}$$

Once the cost are fully covered ($cost_{h,cca_m} = 0$) the household undertakes the chosen action. Adaptation actions are active since the following step and are permanent, except *dry-profing* that expires after $\eta_{dry} > 0$ years[14].

In addition to protective CCA actions to their properties, households can also buy insurance, which upon the payment of an annual premium, refunds to the households the damages experience (or part of it, for more detail about the insurance market, see Subsection "Insurance").

Firms

An important novelty in this version of the CRAB model is that all the sectors combine labor and capital with constant returns to scale to produce a homogeneous product. On the one hand, capital-good firms employ capital and labor to produce capital goods (machines) sold to other firms and invest in R&D to discover more productive technologies. On the other hand, service- and good- produce a consumption product sold to households. Hence, all the firms operate on the capital, labor, and goods/service markets which are characterized by imperfect information. The number of firms is variable and depends on two independent entry and exit processes. Firm might be expiosed to flood and can buy insurance to protect themselves.

Capital-good sector and technological learning

The technology of each capital-firm i is captured by two labor productivity coefficients, A_i^T, and B_i^T. The former coefficient indicates the productivity of the machines that are produced by the firm i and sold to other firms, while the latter stands for the productivity of the firms itself, and it depends on a heterogenous vintage of machines bought from other capital-good firms[2] (for more information about the capital market, see Subsection "Capital").

Capital-good firms determine their price p_i applying a fixed markup ($\mu_1 > 0$) to their unit cost c_i[3]:

$$p_i(t) = (1+\mu_1)c_i(t). \tag{18}$$

The unit cost c_i is the ratio between individual nominal wage w_i and its productivity coefficient:

$$c_i(t) = \frac{w_i(t)}{B_i^T}. \tag{19}$$

Capital firms aim to improve the productivity of the machines they sell (A^T) via technological learning. To do so, they actively invest in R&D a fraction ν_1 of their past sales:

$$R\&D_i(t) = \nu_1 S_i(t-1) \quad with \quad 0 < \nu_1 < 1. \tag{20}$$

Furthermore, firms split their R&D between innovation (IN) and imitation (IM) according to the parameter $\xi \in [0,1]$. Both innovation and imitation are modeled by employing a two-step procedure. In both cases, the first step determines whether innovation or imitation is successful through a draw from a Bernoulli distribution:

$$\theta_i^{in}(t) = 1 - e^{-\zeta_1 IN_i}(t), \tag{21}$$

[2] We assume that capital-good firms cannot self-produce the capital they need for themselves, but they need to order it from other capital-good firms.

[3] Survey data evidence show that European firms mostly set prices according to mark-up rules[15].

$$\theta_i^{im}(t) = 1 - e^{-\zeta_2 IM_i}(t), \quad (22)$$

where $0 \leq \zeta_{1,2} \leq 1$ capture the *search capabilities* of firms. The probability of a positive outcome depends on the amount of resources invested.

Successful firms proceed to the second step. If the innovation draw (Eq.21) is successful, the firm discovers a new technology, (A_i^{in}), according to:

$$A_i^{in}(t) = A_i(t)(1 + x_i^A(t)), \quad (23)$$

where $x^A(t)$ is an independent draw form a $Beta(\alpha_1, \beta_1)$, over the support $[\underline{x_1}, \overline{x_2}]$, with $\underline{x_1} \in [-1, 0]$ and $\overline{x_2} \in [0, 1]$. The supports of the Beta distribution determine the probability of 'successful' over 'failed' innovations, and hence shape the landscape of *technological opportunities*.

Furthermore, firms passing the imitation draw (Eq.22) get access to the technology of one competitor (A_i^{im}). Notably, firms are more likely to imitate competitors with similar technology, and we calculate the technological distance between every pair of firms using a Euclidean metric.

Once both processes are completed, all the firms succeeding in either imitation or innovation select the most efficient production technique they can master according to the following payback period rule (see Subsection "Firms in the consumption-good and consumption-service sectors"):

$$min[p_i^h(t) + bc^h_{A_i(t)}] \quad h = T, in, im \quad (24)$$

where b is a positive payback period parameter (see Eq.32).

Firms in the consumption-good and consumption-service sectors

"Firms in the consumption-good and consumption-service sectors"

Each consumption firm is characterized by heterogenous vintages of capital-goods with different average productivity (A_s), for more information about the capital market see Subsection "Capital", which reflects in its unit cost of production (c_s):

$$c_s(t) = \frac{w_s(t)}{A_s}, \quad (25)$$

where w_s is the average wage paid by firm j. The prices in the consumption-good sector are computed applying a *mark-up* ($\mu_{2,s}$) on unit cost:

$$p_s(t) = (1 + \mu_{2,s})c_s(t). \quad (26)$$

The evolution of firm's market share (f_s), determines the variation of its markup ($\mu_{2,s}$):

$$\mu_{2,s}(t) = \mu_{2,s}(t-1)(1 + \nu \frac{f_s(t-1) - f_s(t-2)}{f_s(t-2)}) \quad with \quad 0 \leq \nu \leq 1. \quad (27)$$

The profits of consumption firms are given by:

$$\Pi_s(t) = (S_s(t) - c_s(j)Q_s(t) - rDeb_s(t), \quad (28)$$

where $S_s(t)$ are the sales of the firm, Q_s is the quantity produced, Deb is the stock of debt and r is the interest rate. Finally, firm liquid assets $NW_s(t)$ are updated according to:

$$NW_s(t) = NW_s(t-1) + \Pi_s(t) - cI_s(t) - cP_s(t), \quad (29)$$

where cI_s and cP_s are the investment and insurance premium costs, respectively.

Flood shock

Firms operating in the regional economy are exposed to flood shocks with different severity and probability (for more information about flood modelling in the CRAB model, see Subsection "Floods"). When a flood does happen, as households, firms calculate their damage coefficient (Dc) overlying flood depth and class-specific depth-damage curve. In this case, we employ the damage coefficients relative to commercial building contents as the main goal of this paper is to analyze the impact of floods on production factors (i.e. destruction of machineries) and inventories. Thus, for firms, we omit the direct damages to buildings. Specifically, floods affect firms via multiple channels:

- A *productivity* shock, which decreases firms' labour productivity for one period: $AB_f(t) = AB_f(t-1)(1 - Dc_f(t))$.
- A *capital stock* shock that destroys a fraction $Dc_f(t)$ of the stock of machines employ by firms.
- An *inventories* shocks that causes a permanent destruction of a fraction of the inventories i.e. $INV_f(t) = INV_f(t-1)(1 - Dc_f(t))$.

Firms entry and exit

In this version of the CRAB model, firms' entry and exit processes are independent. At the end of each period, consumption firms with (quasi) zero market shares and capital-good firms with negative net assets go bankrupt and are removed from the simulation. Conversely, we model the entry process at the microeconomic level, following empirical evidence that shows spillovers as an essential component of agglomeration dynamics[16,17]. More concretely, we assume that if firms make profits above a certain threshold ($\pi_f > q$, where $q = \varepsilon w_f$ with $\varepsilon > 1$), for $\iota > 1$ periods, an employee will open its firm to join the profitable market. Note that if the count is restarted in a period $\pi_f < q$. In line with the empirical findings on firm entry[18,19], we assume that entrants are on average smaller than incumbents. In particular, the stock of capital of new consumption-good firms is equal to a draw from a Uniform distribution with support $[\phi_1, \phi_2]$, with $0 < \phi_1, < \phi_2 \leq 1$, multiplied by the average stocks of the incumbents. Similarly, the stock of liquid assets of entrants in both sectors is obtained by multiplying the average stock in the market by a draw from a Uniform distribution with support $[\phi_3, \phi_4]$, with $0 < \phi_3, < \phi_4 \leq 1$. Concerning the technology of entrants, new consumption-good firms select among the most productive machines. Conversely, the technological frontier of new capital-good firms is drawn from a Beta distribution $Beta(\alpha_2, \beta_2)$. The parameters of the latter determine whether entrants enjoy an advantage or a disadvantage over the incumbents.

In a nutshell, a thriving economy brings the number of firms to increase, triggering agglomeration. Conversely, a stagnant economy generates a higher rate of bankruptcy and, consequently, a progressive abandonment of the region.

Markets

In the CRAB model there are four markets namely Capital, Labor, Consumption, and Insurance. The markets evolve endogenously, shaped by interactions among economic agents and act as formal socio-economic institutional framework.

Capital

The capital-good market is characterized by imperfect information[20]. After updating the productivity coefficient of the machines they are selling (A^T, see Eq.24) capital-good firms send a "brochure" containing the price and productivity of their machines to a random sample of potential new clients (NC_i) as well as its historical customers (HC_i).

All the firms employ adaptive demand expectations ($D^e_f = f[D_f(t-1), D_f(t-2), ..., D_f(t-h)]$), desired inventories ($N^d_f$), and the actual stock of inventories (N_f) form the desired level of production (either if it is machined for capital-firms of consumption product for good- and service- sectors):

$$Q^d_f(t) = D^e_f(t) + N^d_f - N_f(t). \tag{30}$$

The latter is constrained by firms' capital stock K_f, with the desired capital stock K^d_s required to produce Q^d_s. Notably, all the three sectors have different capital-output ratios, Ko_{f1}, Ko_{f2}, and Ko_{f3} mimicking the different degrees of capital intensity required to produce goods vs. services. In case $K^d_f(t) > K_f(t)$, the firm calls for a desired expansionary investment such that:

$$EI^d_f(t) = K^d_f(t) - K_f(t). \tag{31}$$

Furthermore, firms undertake replacement investment RI, scrapping machines with an age above $\eta > 0$ and those that satisfy the following *payback rule*[4]:

$$RI_f(t) = \left\{ A^\tau_f \in \Xi_f(t) : \frac{p^*(t)}{c(A_{f,\tau}, t) - c^*(t)} \leq b \right\}, \tag{32}$$

where p^* and c^* are the price and unit cost of production upon the new machines, and $b > 0$ is the payback period parameter. The total replacement investment is then calculated, summing up all the old vintages that satisfy Eq.32. Furthermore, firms compare the "brochures" received by capital-good firms and order the machines with the best quality/price ratio. Notably, firms are financially constrained, and we assume that firms prioritize capital stock expansion to the substitution of old machines if investment plans cannot be fully realized.

[4]This aligns with multiple empirical studies that demonstrate how replacement investment is typically not proportional to the capital stock[21–23].

Notably, firms have to pay their investments and the workers' wages in advance. This is in line with empirical literature[24–26] that shows that capital markets are imperfect. As a consequence, external funds are more expensive than internal ones, and firms may be credit rationed. More specifically, firms finance their investment first by using their stock of liquid assets (NW_s). When the latter does not fully cover investment costs, firms that are not credit-constrained can borrow the remaining part paying an interest rate r up to a maximum debt/sales ratio of $\Lambda > 1$.

Consumption

Consumption-good and -service firms compete in two markets: Domestic (Dom) and Export (Exp). In a generic market m, firm's competitiveness (E_s) depends on its price, which can account for international (τ) transport cost, as well as on the level of unfilled demand (l_s):

$$E_s^m(t) = -\omega_1 p_s^m(t)(1+\tau) - \omega_2 l_s^m(t) \quad with \quad \omega_{1,2} > 0, \qquad m = [Dom, Exp]. \tag{33}$$

In each market (m), the average competitiveness ($\overline{E}^m$) is calculated by averaging the competitiveness of all firms in the corresponding region weighed by their market share in the previous time step:

$$\overline{E}^m(t) = \sum_{j=1}^{F2} E_s^m(t) f_s^m(t-1) \quad with \quad m = [Dom, Exp]. \tag{34}$$

The market shares (f_s) of firms in the three markets evolve according to the quasi-replicator dynamics:

$$f_s^m(t) = f_s^m(t-1)\left(1 + \chi \frac{E_s^m(t) - \overline{E}^m(t)}{\overline{E}^m(t)}\right) \quad with \quad m = [Dom, Exp], \tag{35}$$

with $\chi > 0$, which measures the selective pressure of the market. In a nutshell, the market shares of the less efficient firms shrink, while those of the most competitive ones increase (due to lower prices and less unfilled demand). Firms' individual demand in each market is then calculated by multiplying their market share by the total demand. In the export market, we assume exogenous demand that grows at a constant rate (α): Finally, firm s calculates individual domestic demand (D^{Dom}) by multiplying market shares and aggregate regional consumption in goods (C^{gd}) or services (C^{serv}), depending on its sector (sec) (for more information about aggregate consumption and export calculation see Subsection "Consumption, taxes, and public expenditures"):

$$D_s^{Dom}(t) = C^{sec}(t) f_s^{Dom} \quad with \quad sec = [gd, serv]. \tag{36}$$

In a similar fashion, the firm calculates the demand from export (D^{Exp}) as:

$$D_s^{Exp}(t) = Exp^{sec}(t) f_s^{Exp} \quad with \quad sec = [gd, serv], \tag{37}$$

hence, firm s obtains its total demand (D_s) at time t by summing domestic and export demands:

$$D_s(t) = D_s^{Dom}(t) + D_s^{Exp}(t). \tag{38}$$

Labor

Firms offer heterogeneous wages which depend on their productivity, as well as on regional productivity, inflation, and unemployment:

$$w_s(t) = w_s(t-1)\left(1 + \psi_1 \frac{\Delta AB_s(t)}{AB_s(t-1)} + \psi_2 \frac{\Delta \overline{AB}(t)}{\overline{AB}(t-1)} + \psi_3 \frac{\Delta U(t)}{U(t-1)} + \psi_4 \frac{\Delta cpi(t)}{cpi(t-1)}\right), \tag{39}$$

With $\psi_1 > 0$, $\psi_2 > 0$ and $\psi_1 + \psi_2 \leq 1$ and where AB_s is firm individual productivity, $\overline{AB}$ is the regional productivity, cpi is the regional consumer price index and U is the local unemployment rate.

Interactions in the local labor markets are decentralized. This process allows accounting for unemployment as a genuine structural out-of-equilibrium phenomenon. The labor supply L^S at time t, is equal to the number of households living in the region. The aggregate labor demand L^D is given by the sum of individual firms' labour demand:

$$L^D(t) = \sum_{i=1}^{F1} \sum_{j=1}^{F2} \sum_{l=1}^{F3} L_f^d \quad with \quad f = [i, j, l], \tag{40}$$

where $F1$, $F2$, and $F3$ are the populations of capital-, consumption- and service-good firms located in the region. The labor demand of capital-good firm i (L_i^d) is equal to:

$$L_i^d = \frac{Qo_i(t)}{B_i(t)}, \tag{41}$$

where Qo_i is the quantity ordered to the firm and B_i its productivity. Similarly, the labor demand of consumption firms c (L_c^d) is computed as:

$$L_c^d = \frac{Qd_c(t)}{A_c(t)}, \tag{42}$$

where Qd_i is its production and A_c its average productivity.

The labor market matching mechanism in the region operates as follows:

1. If $L_f^d(t) > n_f(t)$, where $n_f(t)$ is the current labour force of a generic firm f, the firm posts m vacancies on the labour market, with $m = L_f^d(t) - n_f(t)$. Conversely, if $L_f^d(t) < n_f$ the firm fires m employees.

2. Unemployed households have imperfect information and are boundedly-rational: they are aware only of a fraction $\rho \in (0,1]$ of all vacancies posted by the firms in their home region.

3. Unemployed households sorted by education level, select the vacancy with the highest offered wage in their sub-sample, and they are hired by the corresponding firm.

All things equal, higher education households are prioritized in the labor, and therefore they are likely to get higher salaries than the lower educated. The hiring process is completed when either all households are employed, or when firms have hired all the workers they need. In this out-of-equilibrium market there is no market clearing. Hence, the involuntary unemployment and labor rationing are emergent phenomena generated by the CRAB model.

Insurance

In this version of the model, the insurance market is a sub-category of the service market (being one of the industry included in the macro sector of services, to see how this is managed at macro level see subsection "Consumption, taxes, and public expenditures"). During each time step, the market unfolds as follows:

- A central insurer calculated the expected annual damages (EAD) for all the agents exposed to floods:

$$EAD_a(t) = \int_{p_i}^{p_I} p_i D_{i,a}(t) dp \text{ with } D_i = \begin{cases} Hv_a(t)Dc_a(t) \text{ if } a = h \\ (\Xi_a(t)\overline{p_{f1}} + INV_a(t)p_a(t))Dc_a(t) \text{ if } a = f \end{cases}, \tag{43}$$

  where p_i is the probability of flood event i and p_I is the set of events whose probability is refunded by insurance. Note that the insurance has an entry point p_{max} and an exit point p_{min}, meaning that the damages from floods with $p_i \geq p_{max}$ and $p_i \leq p_{min}$ are not covered by insurance. $D_{i,a}$ is the damages that the flood i causes, which, for households, depends on house value, see Eq.6. Whereas the damages to firms are calculated by multiplying the fraction of whole capital stock (Ξ) and inventories (Inv) that would be destroyed by the flood (according to damage coefficient Dc) by their current market prices. Note that for inventories we use the price of the affected firm, p_a, while for machineries we use the average market price in the capital sector, $\overline{p_{f1}}$, at time t.

- The insurer determines the market price of insurance cost (cP) for agent a at time t by adding a fixed markup to EAD:

$$cP_a(t) = EAD_a(t)(1+\delta), \tag{44}$$

  with $\delta > 0$.

- All agents are assumed to be risk-averse. Hence, they will subscribe to the insurance if they have the resources. This assumption is justified by empirical evidence indicating heightened risk aversion following natural disasters, particularly at a localized level[27].

Consumption, taxes, and public expenditures

A region is regulated by a government agent that taxes the profits of firms and income of households at fixed rates and pays subsidies (w^u) to unemployed households. The latter is a fraction of the regional average wage:

$$w^u(t) = \sigma \overline{W}(t), \quad with \quad \sigma \in [0,1], \tag{45}$$

with $\sigma \in [0,1]$. Workers spend a fraction of their income if employed, hence aggregate regional consumption (C) is equal to the sum of individual consumption:

$$C(t) = \sum_{h=1}^{H} c_h(t), \tag{46}$$

Consumption if further divided between goods (C^{gd}) and services (C^{serv}):

$$C^{gd}(t) = \kappa_1 C(t) + \sum_{h}^{H} cost_{h,cca}(t) + \sum_{h}^{H} cost_{h,repair}(t), \tag{47}$$

With $\kappa \in [0,1]$ being the fraction of good-consumption. In addtion, $\sum_h^H cost_{h,cca}$ and $\sum_h^H cost_{h,repair}$ are the sum of all the money spend in structural adaptation and repair at time t, respectively (being the construction sector a sub-category of the macro goods sector). Furthermore, the aggregate demand for services is:

$$C^{serv}(t) = C(t) - C^{gd}(t) + \sum_{a}^{A} cP_a(t) - \sum_{a}^{H} cC_a(t), \tag{48}$$

with $\sum_a^A cP_a(t)$ being the revenues from insurance premiums (for more detail see Subsection "Insurance") and $\sum_a^H cC_a(t)$ all the claims to refund if a flood happened at time step t.

The model respects the national account identity:

$$\sum_{i=1}^{F1} Q_i(t) + \sum_{j=1}^{F2} Q_j(t) + \sum_{l=1}^{F3} Q_l(t) = Y(t) = C(t) + I(t) + \Delta N(t) + EXP(t) - IMP(t). \tag{49}$$

Since there are no intermediate goods and no imports, the sum of values added to both production sectors (Y), equals their aggregate production, which respectively matches the sum of aggregate consumption (C), investment (I), exports (EXP), imports (IMP) and variations of inventories (ΔN).

Export demand exogenously evolves at a fixed rate α_{exp}

$$Exp(t) = Exp(t-1)(1+g), \quad g > 0. \tag{50}$$

As internal demand, also export is divided between goods (Exp^{gd}) and services (Exp^{serv}):

$$Exp^{gd}(t) = \kappa_2 Exp(t), \tag{51}$$

and $Exp^{serv} = Exp(t) - Exp^{gd}(t)$

Model calibration

Floods

CRAB models how agglomeration and economic development evolves in the presence of climate-driven hazards. Here we employ the CRAB model to contextualize the impact of SLR on flood hazards for Shanghai, one of the most exposed delta megacities. To map the flood exposure of our agents, we combine flood maps of the Shanghai metropolitan area and OpenStreetMap (OSM) residential building data in a two-step procedure[9].

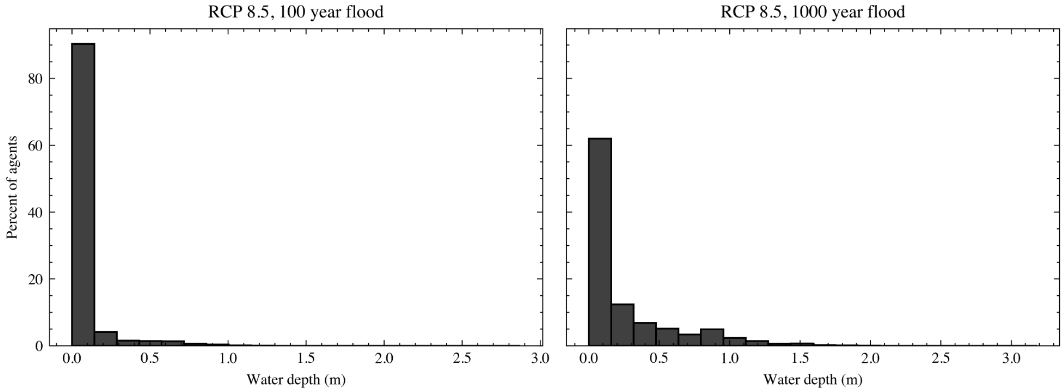

Figure S2. Snapshot of the 100y flood-depth distribution in 2050 in the simulated region. Synthetic flood distribution is generated from[28].

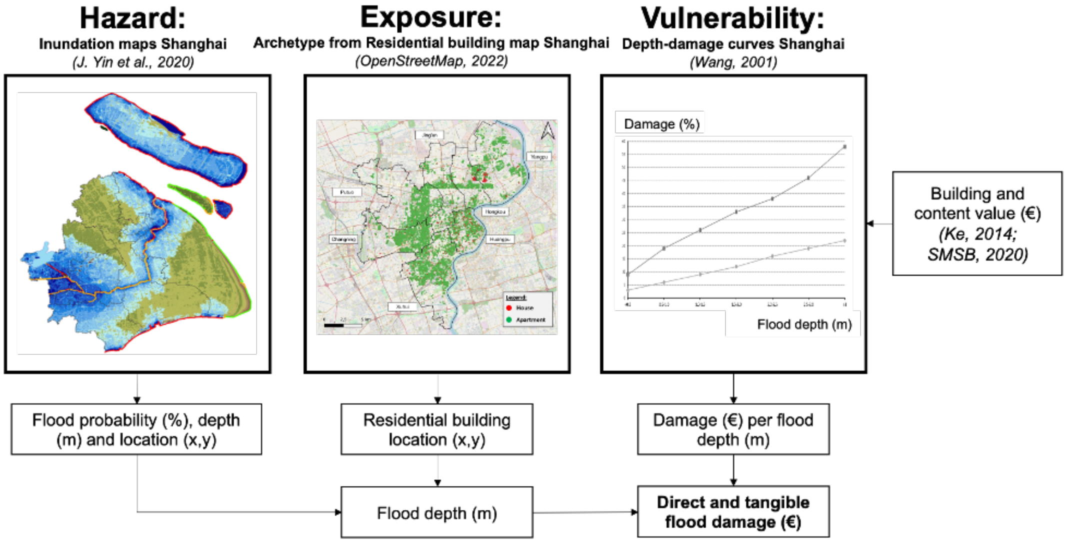

Figure S1. Data for flood risk assessment[9].

First, we create a set of possible locations and related flood depths. The latter includes the 33,374 buildings of the Huangpu, Changning, Yangpu, Xuhui, Jing'an, and Hongkou districts which are around 50% of total residential buildings in OSMs Shanghai. For an example of the resulting synthetic flood distribution see Figure S2. We use the following districts because they provide an accurate representative of survey respondents[5]. Secondly, when an agent is created is randomly assigned to one of these buildings[6]. Notably, following[28], each building has 18 possible flood depths attached to it, which are the ones of three return periods (10y,100y and 1,000y) for three time periods (> 2030, $2030-2050$, $2050-2100$) mapping the worsening of climate conditions and SLRs under two possible scenarios (RCP 2.6 and 8.5) ($3*3*2 = 18$). As a result, when a flood happens, each agent can determine the water level hitting his location depending on the severity of the floods, the time step of the model and the climate change mitigation scenario assumed for that specific model run. Notably, for comparative purposes, we set two fixed floods with a pre-determined return period of $1,000y$ return at $t = 2050$ and $100y$ return at $t = 2060$, conversely $10y$ floods happen stochastically (with an average probability of 1 in 40 steps, i.e. 10 years of model simulation).

[5] These districts are mainly located in the city center of Shanghai which is more exposed to flooding than the rest of the city. However, the data do not include rural and peri-urban areas of the region, which are the most exposed to sea level rise and exacerbating hazards[28]. Hence, we can expect our model to provide an average flood depth that is higher than the city of Shanghai but lower than the surrounding region. Overall, we believe it does not represent a problem with our work as we aim to infer general results for an archetype of a fast-growing economy highly exposed to flood and sea level rise, rather than deriving conclusions from an empirical case study.

[6] Due to limit in data availability, residential building are also assigned to firms.

Survey methodology

Table S1 compares the two "background" socio-economic variables included in the analysis and age. In general, the survey sample is representative of the population. In China many elderly people live with their children or younger family members. As our objective is to study adaptation at the household level, and only one member per household was allowed access to our survey, the lack of older respondents from this country was anticipated, and we do not regard it as problematic for our analysis. In addition, respondents in our sample are more educated than the general population. Importantly, Education is not correlated with any of the other households attributes (see Figure S4). Hence, we calibrate our initial population with the education census data.

Table S1. Comparison of socio-economic variables between Census and survey data.

	Survey (n = 731)		Census	
Variable	*Cat.*	Values	*Cat.*	Values
Gender	Male	52%	Male	50%
	Female	48%	Female	50%
Age	16-24	19%	$\leq$ 17	12%
	25-34	50%	18-34	16.%
	35-44	23%	35-59	37%
	45-54	2%	60+	35%
	55-64	2%		
	65+	1%		
Education	$\leq$ High School	0.4%	$\leq$ High School	47%
	High School	2%	High School	19%
	College degree	69%	College	34%
	Post Graduate	29%		

Given the diminished significance of risk-reducing measures for participants residing in high-rise apartment buildings, we took an additional step to specifically assess the adaptation tendencies of households located on the ground floor ($n = 141$). It's worth highlighting that since our model simulates the first flood occurrence after a span of 50 years (equivalent to 200 time steps), there is no discernible variation in the adoption of wet- and dry-proofing measures (Figure S2.b and S2.c). Nevertheless, based on our projections, there's an anticipated 10% surge in the number of agents affected who might contemplate elevating their dwellings (Figure S2.a). However, high-rise structures aren't necessarily exempt from flood threats. Often, essential infrastructures like boilers, elevators, generators, and water pumps are situated underground in such buildings, making them vulnerable during flood events. Safeguarding these systems is paramount to avert significant damages and disruptions. Moreover, when considering the elevation of a multi-story edifice, the decision typically rests with the building proprietor or the property management team, not exclusively with the residents of the ground floor. In light of these considerations, and given that our complete survey data offers a six-fold increase in data points, we opted to utilize the full dataset. We believe this offers a more nuanced and authentic portrayal of household adaptation responses.

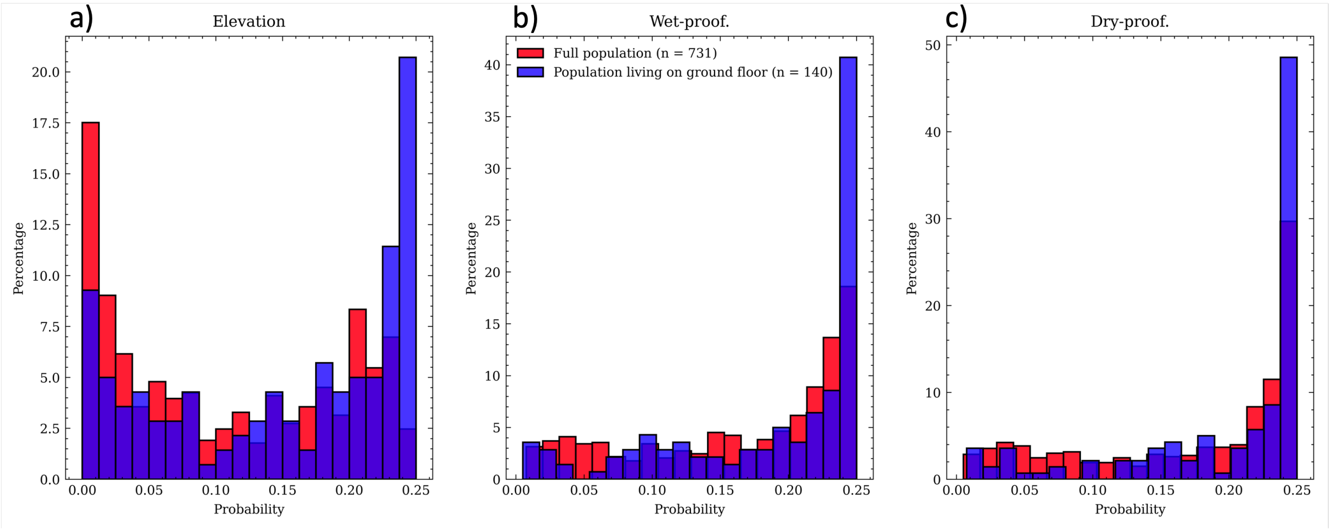

Figure S3. Probability distribution of agents living on the ground floor against the whole population.

Table S2. Socio-behavioral factors of private adaptation intentions. Source: 2020 households' survey in Shanghai, China (N= 731).[13]

Attribute		Mean (std. dev)	Question	Scale
Flood probability		0.12 (0.15)	Imagine you stay in your house for the next 30 years what is the likelihood you believe your household will experience a flood?	0-100%
Worry		2.04 (0.98)	How worried or not are you about the potential impact of flooding on your home?	From 1 (not at all severe) to 5 (very severe)
Response efficacy			How effective do you believe that implementing this measure would be in reducing the risk of flood damage to your home and possessions?	From 1 (extremely ineffective) to 5 (extremely effective)
	Dry-proof.	3.48 (0.89)		
	Wet-proof.	3.44 (0.88)		
	Elevation	3.15 (1.11)		
Self efficacy			Do you have the ability to undertake this measure either yourself or paying a professional to do so?	From 1 (I am unable) to 5 (I am very able)
	Dry-proof.	2.50 (1.16)		
	Wet-proof.	2.49 (1.12)		
	Elevation	2.00 (1.17)		
Perceived costs			When you think in terms of your income and your other expenses, do you believe that implementing (or paying someone to implement) this measure, would be cheap or expensive?	From 1 (very cheap) to 5 (very expensive)
	Dry-proo	3.23 (0.81)		
	Wet proof.	3.35 (0.75)		
	Elevation	3.80 (1.08)		
Flood experience		0.16 (0.3)	Have you ever personally experienced a flood of any kind?	0 - No<br>1 - Yes
Social expectations (Injunctive social norm)		1.67(1.89)	Do your family, friends and/or social network expect you to prepare your household for flooding?	From 1 (do not expect) to 5 (strongly except)
Social network (Descriptive social norm)		1.69 (1.89)	Thinking about your friends, families, and neighbours, how many households have taken some adaptation action towards flooding?	0-8
Undegone (UG) measure			Please indicate if you have already implemented any of these structural measures or if you intend to do so in the future:	0-8
	Dry-proof.	0.13 (0.48)	Installing anti-back flow valves on pipes; Installing a pump and/or one or more system(s) to drainflood water; Fixing water barriers"(e.g., water-proof basement windows).	
	Wet-proof.	0.16 (0.56)	Strengthen the housing foundations to withstand waterpressures; Reconstructing or reinforcing the wallsand/or the ground floor with water-resistant materials; Raising the electricity meter above the mostlikely flood level or on an upper floor.	
	Elevation	0.05 (0.20)	Raising the level of the ground floor above the most likelyflood level.	
Education		3.179 (0.51)	What is the highest level of education you have completed?	From 0 (Less than secondary education) to 4 (Post graduate degree)
House value		3,380,646 (2,603,162)	If you were to put your accommodation on the market today, how much do you believe it would sell for? Please provide your best estimation in the full amount	Open choice (in RMB)
Savings		2.859 (1.452)	With regards to your household's savings, what statement most closely reflects your current household situation?	From 0 (We use practically all of the money we earn each month) to 4 (My household has 4 or more month's wages in savings)

Table S3. Effect sizes of socio-behavioral factors of households' adaptation to climate-driven floods, estimated using logistic regression from the survey data (N=731)

	Elevation (Pseudo R-squared = 0.39)		Wet-proof. (Pseudo R-squared = 0.43)		Dry-proof. (Pseudo R-squared = 0.41)	
	Coeff	Std err.	Coeff	Std err.	Coeff	Std err.
Intercept	-3.96***	0.94	-3.71***	0.97	-7.03***	1.05
Flood damage	0.564***	1.21	0.208*	0.55	0.50**	1.07
Flood probability	0.29	0.70	0.33	0.77	-0.18	0.70
Worry	1.41***	0.37	0.50***	0.12	1.46***	0.42
Flood damage * Worry	-1.54***	0.56	\	\	-1.42***	0.57
Response efficacy	0.15	0.10	0.14**	0.13	0.16	0.13
Self efficacy	0.64***	0.09	0.83***	0.11	0.93***	0.11
Perceived costs	-0.83***	0.11	-0.44***	0.14	0.09	0.60
Flood experience	0.47**	0.26	0.27	0.27	0.41	0.37
Social expectations	0.57***	0.12	0.45***	0.11	0.54***	0.12
Social network	0.26***	0.06	0.37***	0.07	0.55***	0.08
UG Dry-proof.	-0.74**	0.39	-0.74	0.50	\	\
UG Wet-proof.	-0.91**	0.39	\	\	-0.28	0.41
UG Elevation	\	\	-1.67***	0.46	-1.42***	0.54

Households

We utilized the comprehensive behavioral and socio-economic survey data from the municipality of Shanghai ($n = 731$)[137] to formulate a synthetic population of households. To construct a set of agents that reflects the diversity in socio-economic and behavioral characteristics found in the survey, we adopted the following algorithm to sample individual attributes from the empirical data. Table S2 provides summary statistics for both socio-economic and behavioral attributes.

Algorithm 1 Pseudo-code representing the generation of synthetic population of households from empirical data

for $attributes = 1,\ldots,N$ **do**
  Get the *empirical distribution* of the attribute from the survey data
  if I have sampled other attributes before **then**:
    for $sampled \quad attributes = 1,\ldots,N-n$ where n are attributes still to be sampled **do**
      is the distribution of the previously *sampled attribute* correlated with *empirical distribution*?
      if Yes **then**
        empirical distribution→ *empirical distribution* | *sampled attribute*
      end if
    end for
  end if
  random sample a value from the *empirical distribution*
end for

The cross-correlation among variables and mean difference have been used to evaluate criteria between the populations, which are summarized in Fig. S4-S5 and Table S4.

[7]The reference pertains to the utilization of the same dataset, rather than the specific statistical method used.

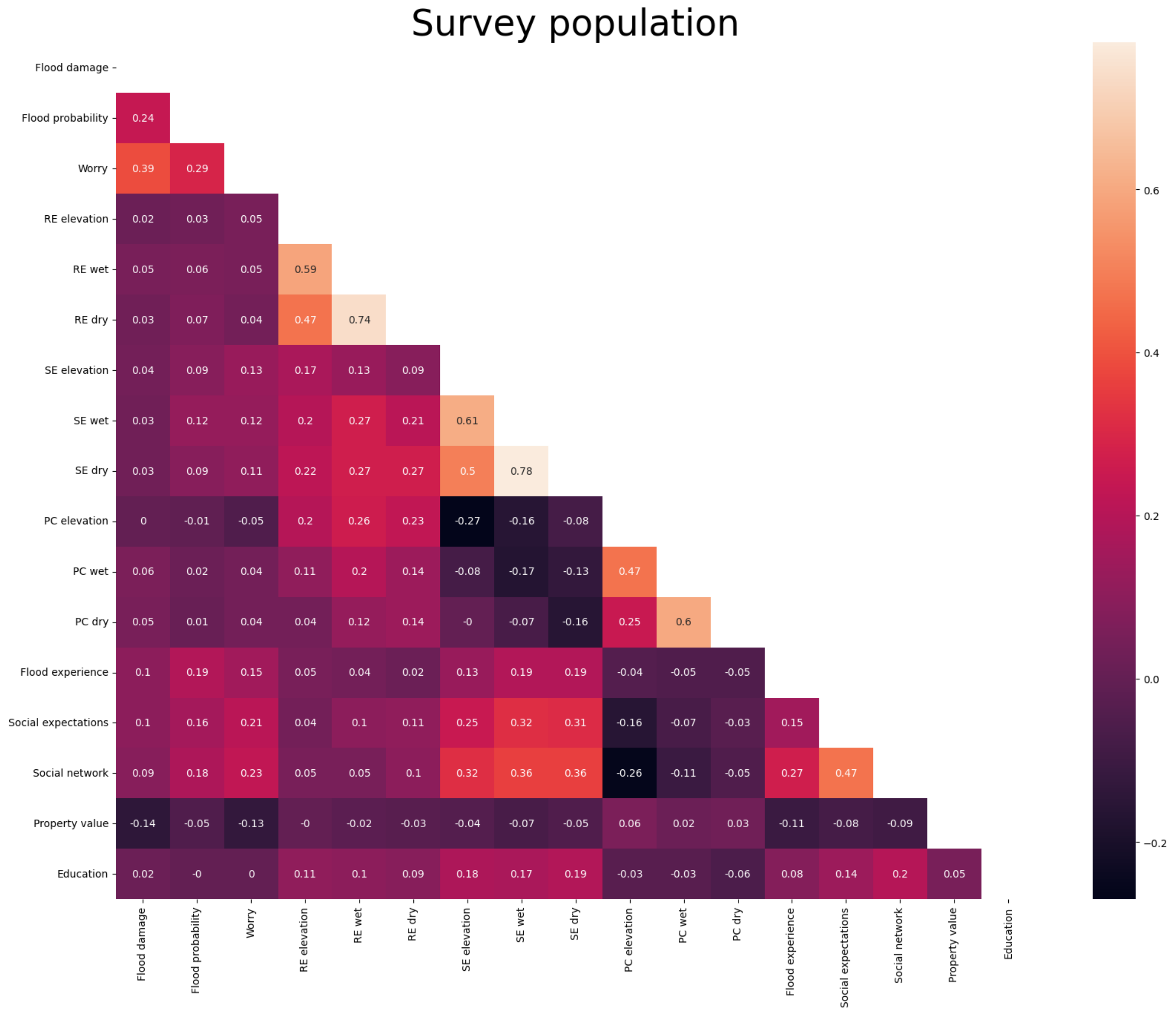

[H]

Figure S4. Pearson correlation among variables from the survey population.

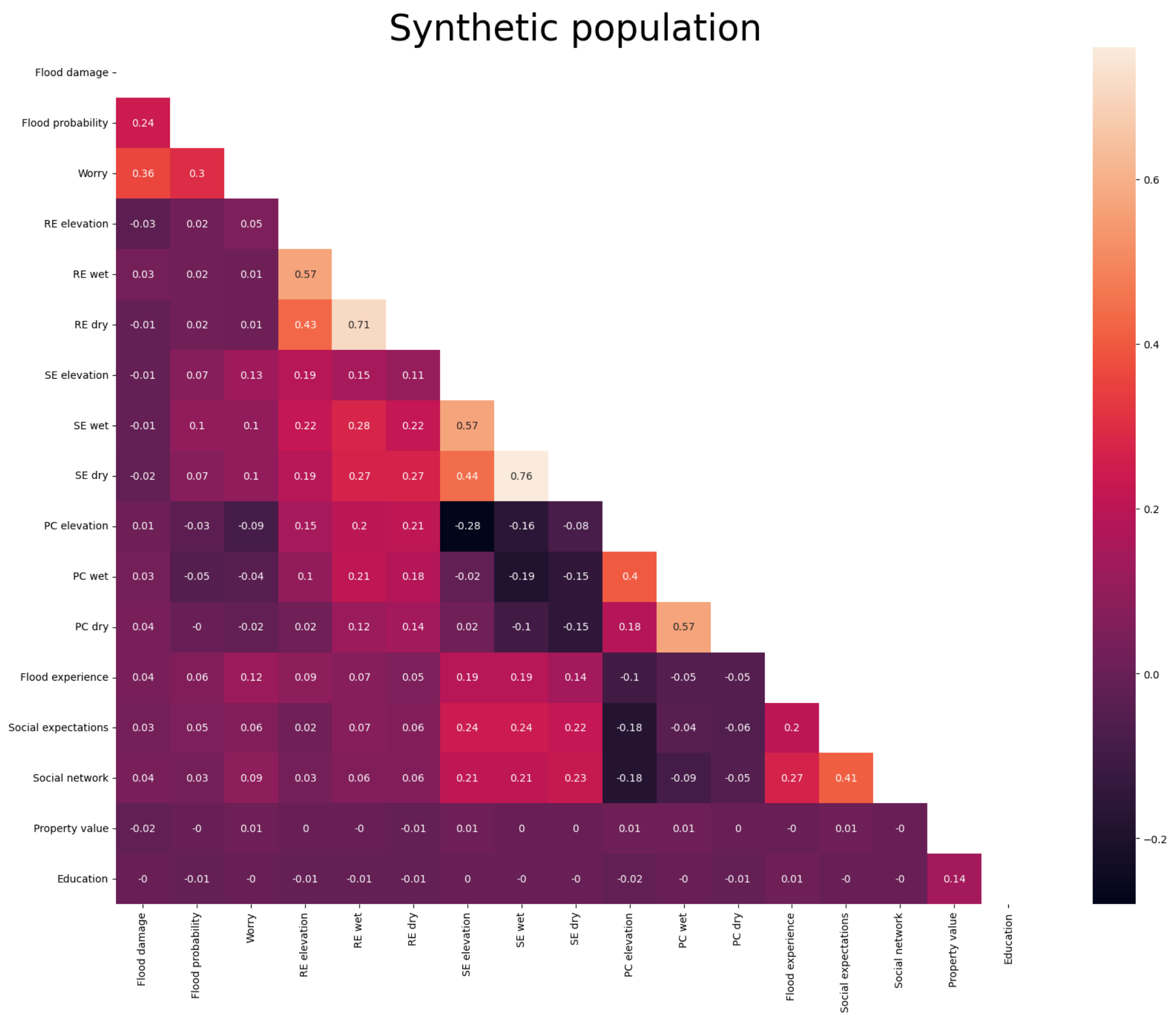

[H]

Figure S5. Pearson correlation among variables from the synthetic population.

Table S4. Mean difference between households survey and synthetic populations

Variable		Mean difference (Survey - Synthetic)
1. Flood damage		0.00
2. Flood probability		-0.01
3. Worry		0.01
4. Response efficacy		-0.03
	Dry-proof.	-0.01
	Wet-proof.	0.02
	Elevation	-0.03
5. Self efficacy		
	Dry-proof.	0.03
	Wet-proof.	0.03
	Elevation	0.03
6. Perceived cost		
	Dry-proof.	0.01
	Wet-proof	-0.01
	Elevation	-0.02
7. Flood experience		0.01
8. Social expectations		0.04
9. Social network		0.06

Climate Change Adaptation actions

We selected seven structural CCA measures from our survey data and grouped them in three categories: *Dry-* and *Wet-proofing*, *Elevation*. For each category, we estimated cost and protection by averaging past literature from the Global North[11]. The related inputs for the current work are summarized in Table S5.

Following FEMA's recommendation, we assume a fixed *Elevation* of 1 meter above the ground[29]. Therefore, households that elevate their property subtract 1 meter from the water depth at their location and are affected by the corresponding damages as per the depth-damage curve. In the context of *Wet-proofing*, we apply a 40% effectiveness rate in reducing both building and content damage[11,30,31]. In determining the effectiveness level, we adhere to the body of work assuming that households typically place valuable goods on the second floor, approximately 3 meters high[32,33].

Dry-Proofing involves a different approach, with an effectiveness value of 85%, if the water level remains below 1 meter, a threshold widely employed in the literature[32–34]. However, if the water level is above one meter, the measure is overtopped, and it has no effectiveness. This aligns with empirical evidence highlighting that *dry-proofing* walls above a specific level may be counterproductive since the pressure difference between the external water and the lack of water inside the building could render it structurally unstable, potentially leading to the failure of the outer walls[11,32].

The application of these data has inherent limitations. The effectiveness of the measures is strongly influenced by local flood conditions[11]. Furthermore, the values we selected are primarily derived from studies conducted in Europe and North America, and may not translate directly to our context in Shanghai city. Local building conditions, content value, and the specific damages caused by floods might vary significantly, and these factors should be taken into consideration in the interpretation of our findings.

Table S5. Grouping, costs, and efficacy of structural CCA measures in the CRAB model, estimate from Survey data[10], n =

Structural CCA measure	Cateogry	Cost measure ($)	Cost category ($)	Protection
1. Installinganti-backflow valves on pipes		240		
2. Installing a pump and/or one or more system(s) to drain flood water	Dry-proofing	443	1,313	85 % d < 1m<br>0% d > 1
3. Fixingwater barriers (e.g.water-proof basement windows)		630		
4. Strengthen the housing foundations towithstand water pressures		1572		
5. Reconstructingor reinforcing the walls and/or the ground floor with water-resistant materials	Wet-proofing	1203	3,226	40%
6. Raisingthe electricity meter above the most likely flood level or on an upper floor		485		
7. Raising the level of the ground floorabove the most likely flood level	Elevation	4,040	4,040	d = d - 1m

Regional economy

We calibrate the CRAB model economy to a stylized agglomerated coastal region, to loosely resemble the greater Shanghai area, in two ways. First, we employ publicly available flood data to determine 30 years' cumulative flood probability and exposure. In a similar fashion, we use national statistics to determine household expenditure on goods and income as well the relative capital intensity of each economic sector. These parameters are summarized in Table S6.

Table S6. Empirically funded parameters in the CRAB model regional economy.

Parameter	Value	Source
Properties Dry-proofed at initialization	0.08	13
Properties Wet-proofed at initialization	0.09	13
Properties Elevated at initialization	0.05	13
Properties Insured at initialization	0.13	13
Intention-behavior gap Φ	0.25	13
Dry-proofing measure lifetime (η_{Dry})	20	10
Households expenditure for goods (% of total consumption, k_1)	35%	35
Households expenditure for services (% of total consumption)	65%	
Capital-output ratio consumption-goods firms (Ko_{gd})	0.7	36
Capital-output ratio consumption-services firms (Ko_{serv})	1.3	

When we refer to the model as being "loosely calibrated", we emphasize the nature of our study, which aims to construct an archetype of a resource-rich coastal megacity rather than an empirical representation of a specific location. Hence, in line with methodologies commonly adopted in agent-based macro-modelling, we tailored the remaining parameters of the CRAB model's regional economy to emulate six empirical attributes of real-world systems[37,38]. At its core, the indirect calibration process identifies particular empirical features for the model to replicate. This involves utilizing a search strategy, frequently employing Monte Carlo sampling, to pinpoint suitable parameter values. This is followed by a rigorous assessment of the robustness of these chosen conditions in the simulated outputs by considering nearby parameter values and altering the pseudo-random number generator's seed. In particular, the six conditions to be satisfied by the simulated data are:

- Pattern of self-sustained growth with persistent fluctuations.
- Average annual growth rate for output around 3%.
- Average unemployment rate between 5% and 15%.
- Output is less volatile than investment and more than consumption.
- Technological innovation generates agglomeration.
- Floods decrease entry and exit of firms.

The parameters from the indirect calibration approach are summarized in Table S7.

Table S7. Past literature and indirect calibration parameters in the CRAB regional economy

Description	Symbol	Value
Number of firms in capital-good industry	F_1	250
Number of firms in consumption-good industry	F_2	400
Number of firms in consumption-service industry	F_3	600
Number of households	H	$10,000$
R&D investment propensity	ν	0.04
R&D allocation to innovative search	ξ	0.5
Firm search capabilities parameters	$\zeta_{1,2}$	0.3
Beta distribution parameters (innovation process)	(α_1, β_1)	$(2,4)$
Beta distribution support (innovation process)	$[\underline{x_1}, \overline{x_2}]$	$[-0.05, 0.05]$
Profits to wage ratio	ε	2
Consecutive number of periods for new firms creation	ι	6
New-customer sample parameter	γ	0.2
Capital-good firm mark-up rule	μ_1	0.15
Desired inventories	l	0.1
Payback period	b	3
"Physical" scrapping age	η	20
Mark-up coefficient	υ	0.04
Competitiveness weights	$\omega_{1,2}$	1
international transport cost	τ	0.06
Replicator dynamics coefficient	χ	1
Maximum debt/sales ratio	Λ	2
Interest rate	r	0.01
Uniform distribution supports (consumption-good entrant capital)	$[\phi_1, \phi_2]$	$[0.10, 0.90]$
Uniform distribution supports (entrant stock of liquid assets)	$[\phi_3, \phi_4]$	$[0.10, 0.90]$
Beta distribution parameters (capital-good entrants technology)	(α_1, β_2)	$(2,4)$
Wage setting $\Delta\overline{AB}$ weight	ψ_1	0.2
Wage setting ΔAB_i weight	ψ_2	0.8
Wage setting Δcpi_r weight	ψ_3	0
Wage setting ΔU_r weight	ψ_4	0
Labour search sample parameter	ρ	0.3
Tax rate	tr	0.2
Insurer mark-up	δ	0.05
Mix balance parameter	o	0.3
Minimum unemployment for exit	U_{min}	0.05
Maximum unemployment for entry	U_{max}	0.2
Unemployment subsidy rate	σ	0.5
Export growth rate	g	0.01

Model validation

We validate the model according to its ability to reproduce a wide ensemble of micro and macro stylized facts, which are summarized in Table S8. For additional detail about the CRAB model validation see[39].

Table S8. Key economic empirical stylized facts replicated by the model.

Stylized facts (SF)	Empirical studies
**Flood related aggregate-level stylized facts**	
SF1 Flood decreases economic output	40,41
SF2 Flood decreases employment	41,42
SF3 Flood decreases entry of firms	43
**Region economy aggregate-level stylized facts**	
SF4 Endogenous self-sustained growth with persistent fluctuation	44–46
SF5 Relative volatility of GDP, consumption, investments	44,47
SF6 Cross-correlations of macro-variables	44,47
SF7 Pro-cyclical aggregate R&D investment	48
SF8 Persistent unemployment	49–51
**Region economy firm-level stylized facts**	
SF9 Not all firms export	52,53
SF10 Exporters are more productive and larger than non-exporters	52,53
SF11 Firm (log) size distribution is right-skewed	54
SF12 Productivity heterogeneity across firm	19,54,55
SF13 Persistent productivity differential across firm	19,54,55
SF14 Lumpy investment rates at firm level	56

Sensitivity analysis

We apply the one-factor-at-a-time (OFAT) sensitivity analysis, where a single parameter is adjusted while the rest are held constant to examine the potential variability in results[57]. The OFAT method was chosen for its lower computational demands in comparison to more intensive techniques such as variance decomposition[58]. Moreover, while comprehensive sensitivity analysis methods can sometimes overlook the complexities of intricate dynamics and emergent behaviors typical of agent-based models, the OFAT approach provides better insight into such dynamics[59]. We then measure the influence of these parameter changes on our primary outcome: difference in economic growth and development across specific scenarios.

Considering the architectural landscape of many cities, where a significant portion of the population resides in high-rise buildings, our methodology might present a skewed estimation of households vulnerable to flooding. For instance, our findings suggest that around 20% could be exposed to a 100-year flood by 2050 (refer to Figure S2). As a result, we employed the OFAT SA on the percentage of households exposed, utilizing our baseline (20% for a 100-year flood in 2050) as the upper limit, and then progressively reducing this exposure down to zero. While the proportion of households affected by flooding influences our results, with more exposure correlating to reduced long-term economic growth, the overarching insights remain consistent. This consistency arises primarily from the economic upheaval triggered by the disruption to firms, which subsequently generates indirect economic implications on households (for an in-depth exploration of the different channels affecting the economy in the CRAM model individually, see[39]). Our investigations bolstered our initial conclusions: integrating bottom-up CCA with overarching subsidies generates the most robust societal frameworks to withstand climate adversities. Sole reliance on autonomous measures falls short in countering severe climatic incidents. Furthermore, the average adverse outcomes experienced by households persist, with those in the lower-income bracket disproportionately facing the indirect aftermath.

Additionally, recognizing the pivotal role of migration parameters and the unpredictability they introduce, we undertook a sensitivity analysis on the balance parameters (o) and the unemployment thresholds required for either the entry or the exit of households (U_{min} and U_{max}, Eq.1, see Table S7 for original values). The oscillations in the scale of results, however, did not alter the central conclusion: the repercussions of inaction in the face of a potential flood are distinct and significant ('None' scenario in Figure S7). Yet, when juxtaposing the outcomes of implementing bottom-up CCA measures and the top-down subsidy against the baseline scenario, the difference is often subtle, underscoring that the combination of such measures bears similar outcomes to the baseline conditions in terms of economic growth and development ('Subsidy & DR & Insurance' in Figure S7).

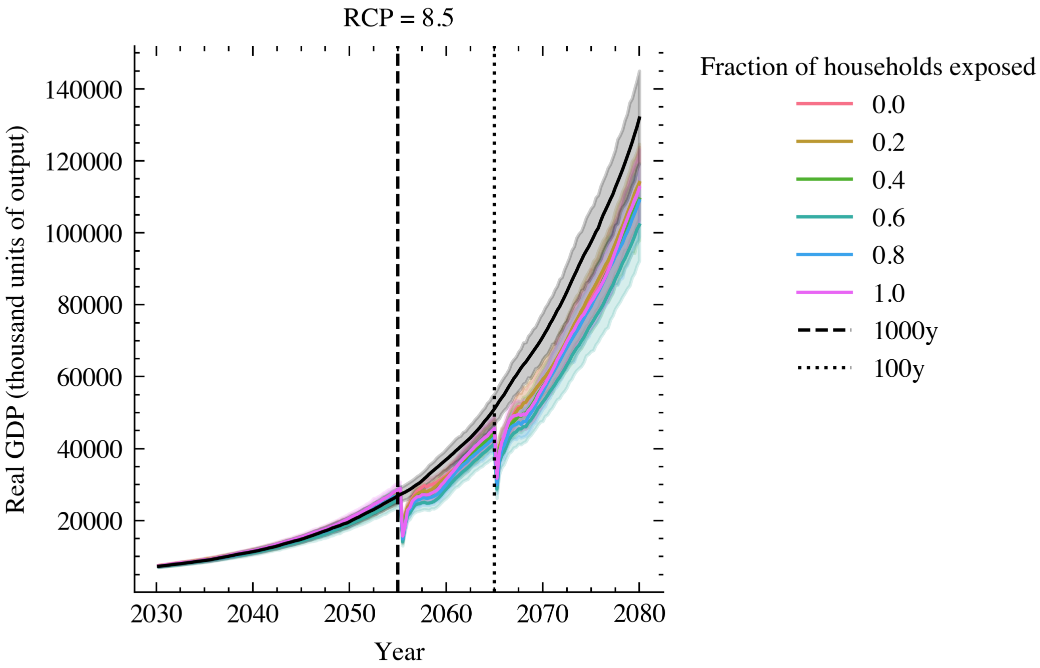

Figure S6. Sensitivity analysis that shows the average economic growth under each different fraction of households exposed to floods. Note that 1 indicated the fraction of households exposed in the Baseline scenario. The reported values are under RCP 8.5 and average across the 100 Monte Carlo runs with the shaded areas denoting the standard deviation.

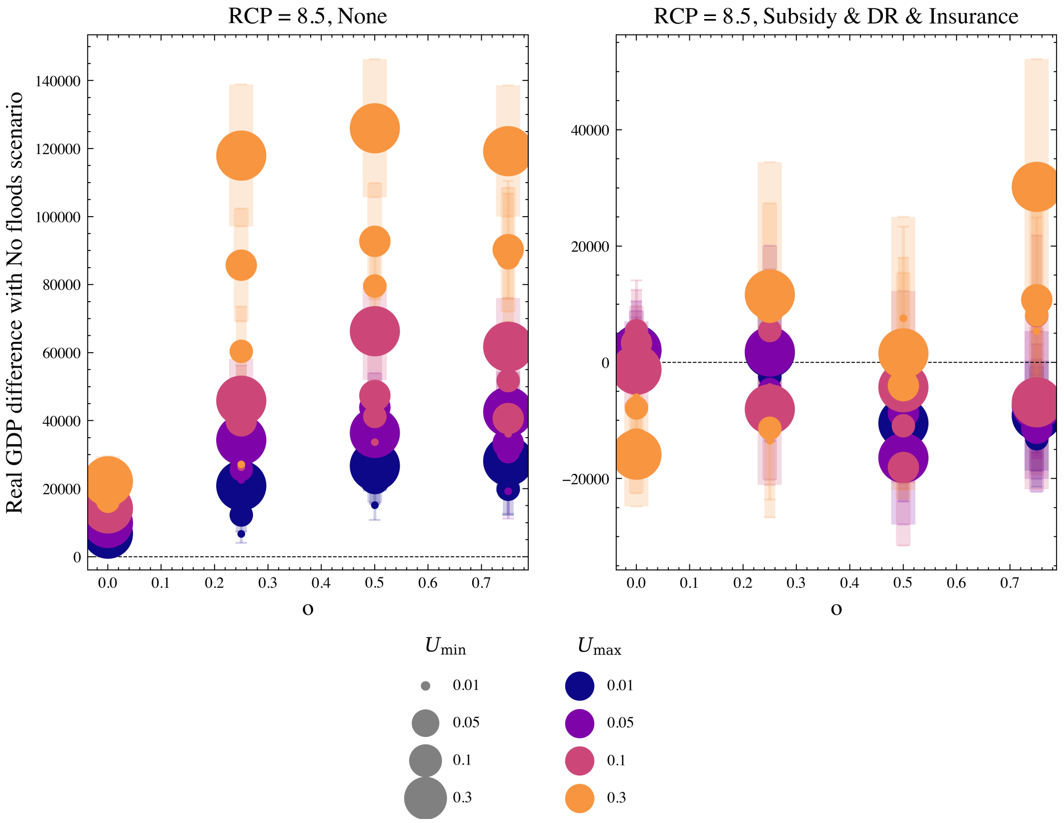

Figure S7. Panel (a) shows the Real GDP (unit of output produced) difference when no adaptation action is taken ('None') compared to the 'Baseline - No flood' scenario. Panel (b) makes the same comparison with the 'Subsidy & DR & Insurance' scenario. The reported values are under RCP 8.5 and average across the 100 Monte Carlo runs. The horizontal dashed line indicated when there is no difference.

References

1. Dosi, G., Fagiolo, G. & Roventini, A. Schumpeter meeting Keynes: A policy-friendly model of endogenous growth and business cycles. *J. Econ. Dyn. Control.* **34**, 1748–1767, DOI: 10.1016/j.jedc.2010.06.018 (2010).
2. Dosi, G., Fagiolo, G., Napoletano, M. & Roventini, A. Income distribution, credit and fiscal policies in an agent-based Keynesian model. *J. Econ. Dyn. Control.* **37**, 1598–1625, DOI: 10.1016/j.jedc.2012.11.008 (2013).
3. Lamperti, F., Dosi, G., Napoletano, M., Roventini, A. & Sapio, A. Faraway, So Close: Coupled Climate and Economic Dynamics in an Agent-based Integrated Assessment Model. *Ecol. Econ.* **150**, 315–339, DOI: 10.1016/j.ecolecon.2018.03.023 (2018).
4. Lamperti, F., Bosetti, V., Roventini, A. & Tavoni, M. The public costs of climate-induced financial instability. *Nat. Clim. Chang.* **9**, 829–833, DOI: 10.1038/s41558-019-0607-5 (2019).
5. Erdős, P., Rényi, A. *et al.* On the evolution of random graphs. *Publ. Math. Inst. Hung. Acad. Sci* **5**, 17–60 (1960).
6. Kennan, J. & Walker, J. R. The effect of expected income on individual migration decisions. *Econometrica* **79**, 211–251 (2011).
7. Dosi, G., Roventini, A. & Russo, E. Endogenous growth and global divergence in a multi-country agent-based model. *J. Econ. Dyn. Control.* **101**, 101–129, DOI: 10.1016/j.jedc.2019.02.005 (2019).
8. Davidoff, T. Labor income, housing prices, and homeownership. *J. urban Econ.* **59**, 209–235 (2006).
9. Lechner, J. Role of household climate change adaptation in reducing coastal flood risk: The case of shanghai (2022).
10. Noll, B., Filatova, T. & Need, A. One and done? exploring linkages between households' intended adaptations to climate-induced floods. *Risk analysis* (2022).
11. Kreibich, H., Bubeck, P., Van Vliet, M. & De Moel, H. A review of damage-reducing measures to manage fluvial flood risks in a changing climate. *Mitig. Adapt. Strateg. for Glob. Chang.* **20**, 967–989, DOI: 10.1007/s11027-014-9629-5 (2015).
12. Rogers, R. W. A protection motivation theory of fear appeals and attitude change. *The J. Psychol.* **91**, 93–114, DOI: 10.1080/00223980.1975.9915803 (1975).
13. Noll, B., Filatova, T., Need, A. & Taberna, A. Contextualizing cross-national patterns in household climate change adaptation. *Nat. climate change* **12**, 30–35 (2022).
14. Du, S. *et al.* Hard or soft flood adaptation? advantages of a hybrid strategy for shanghai. *Glob. Environ. Chang.* **61**, 102037 (2020).
15. Fabiani, S. *et al.* What firms' surveys tell us about price-setting behavior in the euro area. *Int. J. Cent. Bank.* **2**, 3–47 (2006).
16. Bischi, G. I., Dawid, H. & Kopel, M. Spillover effects and the evolution of firm clusters. *J. Econ. Behav. Organ.* **50**, 47–75, DOI: 10.1016/S0167-2681(02)00040-9 (2003).
17. Frenken, K. & Boschma, R. A. A theoretical framework for evolutionary economic geography: Industrial dynamics and urban growth as a branching process. *J. Econ. Geogr.* **7**, 635–649, DOI: 10.1093/jeg/lbm018 (2007).
18. Caves, R. E. Industrial Organization and New Findings on the Turnover and Mobility of Firms. *J. Econ. Lit.* **36**, 1947–1982 (1998).
19. Bartelsman, E., Scarpetta, S. & Schivardi, F. Comparative analysis of firm demographics and survival: Evidence from micro-level sources in OECD countries. *Ind. Corp. Chang.* **14**, 365–391, DOI: 10.1093/icc/dth057 (2005).
20. Phelps, E. & Winter, S. G. Optimal price and advertising policy under atomistic competition. *J. Econ. Dyn. Control.* **4**, 57–71, DOI: 10.1016/0165-1889(82)90003-3 (1982).
21. Feldstein, M. S. & Foot, D. K. The other half of gross investment: Replacement and modernization expenditures. *The Rev. Econ. Stat.* **53**, 49–58 (1971).
22. Eisner, R. Components of capital expenditures: Replacement and modernization versus expansion. *The Rev. Econ. Stat.* **54**, 297–305 (1972).
23. Goolsbee, A. The business cycle, financial performance, and the retirement of capital goods. *Rev. Econ. Dyn.* **1**, 474–496 (1998).
24. Stiglitz, J. E. & Weiss, A. Credit Rationing in Markets with Rationing Credit Information Imperfect. *The Am. Econ. Rev.* **71**, 393–410, DOI: 10.2307/1802787 (1981).

25. Greenwald, B. C. & Stiglitz, J. E. Financial Market Imperfections and Business Cycles. *The Q. J. Econ.* **108**, 77–114, DOI: 10.2307/2118496 (1993).

26. Hubbard, R. G. Capital-Market Imperfections and Investment. *J. Econ. Lit.* **36**, 193–225, DOI: 10.3386/W5996 (1997).

27. Bourdeau-Brien, M. & Kryzanowski, L. Natural disasters and risk aversion. *J. Econ. Behav. & Organ.* **177**, 818–835 (2020).

28. Yin, J. *et al.* Flood risks in sinking delta cities: time for a reevaluation? *Earth's Futur.* **8**, e2020EF001614 (2020).

29. Federal Emergency Management Agency. Fema p-348, protecting building utilities from flood damage. Tech. Rep., U.S. Department of Homeland Security, Washington, D.C. (2017).

30. Egli, T. Non structural flood plain management: measures and their effectiveness. Tech. Rep., International Commission for the Protection of the Rhine (ICPR) (2002).

31. Defra, J. Developing the evidence base for flood resistance and resilience. Tech. Rep., Department for Environment, Food and Rural Affairs London (2008).

32. de Moel, H., van Vliet, M. & Aerts, J. C. Evaluating the effect of flood damage-reducing measures: a case study of the unembanked area of rotterdam, the netherlands. *Reg. environmental change* **14**, 895–908 (2014).

33. Lasage, R. *et al.* Assessment of the effectiveness of flood adaptation strategies for hcmc. *Nat. Hazards Earth Syst. Sci.* **14**, 1441–1457 (2014).

34. Bubeck, P. & de Moel, H. Sensitivity analysis of flood damage calculations for the river rhine. *IVM Rep.* (2010).

35. United States Census Bureau. 2020 census results (2021). Accessed: 2021-08-01.

36. Commission, E., Centre, J. R., Ivanova, O., Kancs, d. & Thissen, M. *EU economic modelling system : assessment of the European Institute of Innovation and Technology (EIT) investments in innovation and human capital* (Publications Office, 2019).

37. Windrum, P., Fagiolo, G. & Moneta, A. Empirical Validation of Agent-Based Models: Alternatives and Prospects. *J. Artif. Soc. Soc. Simul.* **10**, 1–8 (2007).

38. Fagiolo, G., Guerini, M., Lamperti, F., Moneta, A. & Roventini, A. Validation of Agent-Based Models in Economics and Finance. Tech. Rep. (2017).

39. Taberna, A., Filatova, T., Roventini, A. & Lamperti, F. Coping with increasing tides: Evolving agglomeration dynamics and technological change under exacerbating hazards. *Ecol. Econ.* **202**, 107588, DOI: https://doi.org/10.1016/j.ecolecon.2022.107588 (2022).

40. Amin, A. Post-Fordism: Models, Fantasies and Phantoms of Transition. *Post-Fordism* **1**, 1–7, DOI: 10.1002/9780470712726.ch1 (1994).

41. Feldman, M. P. & Kogler, D. F. *Stylized facts in the geography of innovation*, vol. 1 (Elsevier BV, 2010).

42. Thomas, L. F. *The World Is Flat* (Farrar, Straus and Giroux, New York, NY, 2005).

43. Jia, R., Ma, X. & Xie, V. W. Expecting floods: Firm entry, employment, and aggregate implications. *NBER* (2022).

44. Stock, J. H. & Watson, M. W. Chapter 1 Business cycle fluctuations in us macroeconomic time series, DOI: 10.1016/S1574-0048(99)01004-6 (1999).

45. Zarnowitz, V. Recent Work on Business Cycles in Historical Perspective: Review of Theories and Evidence. Tech. Rep., National Bureau of Economic Research, Cambridge, MA (1984). DOI: 10.3386/w1503.

46. Kuznets, S. & Murphy, J. *Modern economic growth: Rate, structure, and spread* (New Haven and London Yale University Press, 1966).

47. Napoletano, M., Roventini, A. & Sapio, S. Are Business Cycles All Alike? A Bandpass Filter Analysis of Italian and US Cycles (2004).

48. Wälde, K. & Woitek, U. R&D expenditure in G7 countries and the implications for endogenous fluctuations and growth. *Econ. Lett.* **82**, 91–97, DOI: 10.1016/j.econlet.2003.07.014 (2004).

49. Blanchard, O. J. & Summers, L. H. Hysteresis and the European Unemployment Problem. *NBER Macroecon. Annu.* **1**, 15–78, DOI: 10.1086/654013 (1986).

50. Blanchard, O. & Wolfers, J. The role of shocks and institutions in the rise of European unemployment: The aggregate evidence. *Econ. J.* **110**, 1–33, DOI: 10.1111/1468-0297.00518 (2000).

51. Ball, L. Hysteresis in Unemployment: Old and New Evidence. Tech. Rep., National Bureau of Economic Research, Cambridge, MA (2009). DOI: 10.3386/w14818.

52. Bernard, A. B. & Durlauf, S. N. Convergence in international output. *J. Appl. Econom.* **10**, 97–108, DOI: 10.1002/JAE.3950100202 (1995).

53. Bernard, A. B., Jensen, J. B., Redding, S. J. & Schott, P. K. The Empirics of Firm Heterogeneity and International Trade. *http://dx.doi.org/10.1146/annurev-economics-080511-110928* **4**, 283–313, DOI: 10.1146/ANNUREV-ECONOMICS-080511-110928 (2011).

54. Dosi, G. Statistical regularities in the evolution of industries: A guide through some evidence and challenges for the theory. In *Perspectives on Innovation*, 153–186, DOI: 10.1017/CBO9780511618390.009 (Cambridge University Press, 2007).

55. Bartelsman, E. J. & Doms, M. Understanding productivity: Lessons from longitudinal microdata. *J. Econ. Lit.* **38**, 569–594, DOI: 10.1257/jel.38.3.569 (2000).

56. Doms, M. & Dunne, T. Capital Adjustment Patterns in Manufacturing Plants. *Rev. Econ. Dyn.* **1**, 409–429, DOI: 10.1006/redy.1998.0011 (1998).

57. Schervish, M. J., Law, A. M. & Kelton, W. D. Simulation Modeling and Analysis. *J. Am. Stat. Assoc.* **78**, 743, DOI: 10.2307/2288169 (1983).

58. Saltelli, A. *et al. Global Sensitivity Analysis: The Primer* (Wiley-Interscience, Chichester, England ; Hoboken, NJ, 2008), 1 edition edn.

59. ten Broeke, G., van Voorn, G. & Ligtenberg, A. Which sensitivity analysis method should i use for my agent-based model? *JASSS* **19**, 20–39, DOI: 10.18564/jasss.2857 (2016).
